# Provision of peer support at the intersection of homelessness and problem substance use services: a systematic ‘state of the art’ review

**DOI:** 10.1186/s12889-020-8407-4

**Published:** 2020-05-07

**Authors:** Joanna Astrid Miler, Hannah Carver, Rebecca Foster, Tessa Parkes

**Affiliations:** Salvation Army Centre for Addiction Services and Research, Faculty of Social Sciences, University of Stirling, Colin Bell Building 4S31, Stirling, FK9 4LA UK

## Abstract

**Background:**

Peer support refers to a process whereby individuals with lived experience of a particular phenomenon provide support to others by explicitly drawing on their personal experience. It has been adopted in a variety of service contexts including homelessness, substance use, mental and physical health. Those who experience homelessness have some of the most complex intersecting health and social challenges. This ‘state of the art’ review provides a systematic search and synthesis of literature examining use of peer support models within services for people impacted by homelessness and problem substance use.

**Methods:**

A systematic search using six databases (CINAHL, SocINDEX, PsycINFO, MEDLINE, Scopus and Web of Knowledge) was conducted in August 2019 and identified 2248 papers published in English after the year 2000. After de-duplication and scanning titles/abstracts, 61 papers were deemed relevant. Three more papers (including one grey literature report) were identified via references, but two papers were later excluded due to relevance. The final 62 papers included studies conducted in five countries. A thematic analysis approach was used to compare and contrast the study findings and provide a synthesis of the main learning points.

**Results:**

In recent years there has been a substantial increase in research examining the utility of peer support yet there is significant variation across this field. Alongside profiling the range of settings, aims, populations, and main outcomes of these studies, this paper also provides an overview of overarching themes: the overall effectiveness and impact of peer-staffed or peer-led interventions; and challenges commonly faced in these roles. Five themes relating to the challenges faced by peers were identified: vulnerability, authenticity, boundaries, stigma, and lack of recognition.

**Conclusions:**

While our findings provide support for current efforts to involve individuals with lived experience in providing peer support to those experiencing concurrent problem substance use and homelessness, they also urge caution because of common pitfalls that can leave those providing the support vulnerable. We conclude that peers should be respected, valued, supported, and compensated for their work which is often profoundly challenging. Suggested guidelines for the implementation of peer involvement in research studies and service delivery are presented.

## Background

Homelessness is a complex term with no uniform definition. It encompasses a wide range of housing situations. These include unsheltered environments such as the streets; and sheltered environments such as in temporary accommodation, or with friends or family [1]. Estimates suggest that 307,000 people in the UK [2], 550,000 in the USA [3], and 35,000 in Canada [4], experience homelessness at any one point, and in recent years these rates have been increasing [2]. It is important to highlight that, due to the variations in the definitions of homelessness, rates may under-represent the true scale. Indeed, with the aim of facilitating more meaningful international comparisons, Busch-Geertsema and colleagues [1] propose a global definition of homelessness where homelessness is conceptualised as ‘*lacking access to minimally adequate housing*’ (p.125).

Those who experience homelessness are often considered to be ‘hard to reach’ and typically experience ‘deep social exclusion’ [5]. People who are homeless usually have some of the most complex intersecting health and social challenges and are vulnerable to ‘tri-morbidity’, the co-occurrence of poor mental health, poor physical health, and problem substance use [6]. These challenges often cause or contribute to breakdowns in relationships with family, friends, as well as breakdowns in contact with support services [7]. In addition to being disproportionately affected by health inequalities [8], people who are homeless tend to experience multiple social challenges such as isolation and feelings of worthlessness, leading to depression and loneliness [9]. They are also at increased risk for developing serious physical illness, such as Tuberculosis (TB), the Human Immunodeficiency Viruses (HIV) / Acquired Immunodeficiency Syndrome (AIDS), Hepatitis (A, B and C), and other infectious diseases [10], and for using alcohol, drugs and tobacco [11]. Taken together, these factors leave this population very vulnerable.

Peer support refers to a process whereby individuals with lived experience of a particular phenomenon provide support to others by explicitly drawing on their experience of this situation. Peer support can be informal, involving ad hoc support from one individual to another; and formal with peers trained to offer support in a structured way. We applied this inclusive definition of peer support to conduct this review. The idea that peers can help others through specific struggles has long been established, with uptake in mental health settings increasing substantially since the 1970s [12]. Peer support has since moved into other service areas including homelessness, criminal justice settings, substance use treatment, and physical health (e.g. [13–15]). Peer support stemmed from the mental health recovery movement which rejected what they considered to be an outdated and stigmatising medical model for mental health treatment [16]. People who understood themselves as psychiatric survivors sought reform on, for example, hospital procedures for those in crisis, as well as greater acknowledgement of the social factors that contribute to distress, and the value of lived experience [12]. Research into peer support in mental health services is increasing [17], with recent reviews suggesting that evidence for effectiveness is mixed [18]. This is partially due to a lack of uniform understanding, definitions, and clearly specified job roles for peers in this arena [18].

In 2003, Wallcraft and colleagues identified over 700 programmes involving peers and consumers in England [19]. Since then the number of peer interventions has continued to increase globally, with interventions found in numerous organisations, across multiple sectors. The value of these interventions is also being increasingly recognised, as reflected in recommendations for peer involvement within international guidelines for a variety of health issues. For example, researchers in Australia have developed recommendations for the use of peer support within ‘high-risk’ environments, where personnel are routinely exposed to potentially traumatic events, such as emergency services and the military [20] and Canadian advisory groups have developed national guidelines on the inclusion of those with lived experience of homelessness services [21]. There is also strategic policy support for peer involvement, evident, for example, in the Scottish national drug and alcohol treatment strategy [22] and in Australia [23] and Canada [24].

Despite the increasing popularity of peer-led and peer-staffed interventions, and the subsequent increase in research on peer involvement, interventions that specifically address the intersection of homelessness and problem substance use have not been systematically reviewed. This ‘state of the art’ review addresses this gap by providing a systematic search and synthesis of literature examining the use of peer support models for people impacted by homelessness and problem substance use. We conclude by presenting a set of guidelines designed to support enhanced transparency of reporting these models and to address the frequently experienced challenges in service settings.

## Methods

### Study design

This was a ‘state of the art review’ which, according to Grant and Booth’s review classification, aims for comprehensive searching of recent literature, addresses more contemporary matters in comparison to other combined retrospective and current approaches, and aims to examine current knowledge, offer new perspectives and highlight avenues for further research [25].

### Search strategy

The review protocol was developed by the first author (JM) and reviewed by all other authors (TP, HC, RF). Any type of article or report that mentioned all three topics of interest were considered: 1) peer support, peer workers, peer mentors, peer advocates, peer educators, peer researchers, people with lived experience; 2) problem substance use (drugs, tobacco and/or alcohol); and 3) homelessness (or being at risk of homelessness, including rough sleeping, hostels, prisons etc.). Studies that were not eligible were missing any of the three above components. We did not set any additional inclusion/exclusion criteria, such as the minimum number of participants identifying as homeless or identifying as substance users in the study samples. While we are aware that this is good practice for systematic reviews, and these criteria have been used in a recent systematic review of the effectiveness of peer support with those who are homeless [26], we chose not to do this due to the fact that a state of the art review aims to capture all the potentially relevant literature published on the topic of interest. We therefore aimed to capture the breadth of evidence across both homelessness and problem substance use fields. We were as inclusive as possible while still having a manageable review by capturing data examining peer support within other contexts that, by chance, had participants experiencing homelessness in their sample. The research spanned the following target population or health condition/at risk groups: people with TB, HIV, or Hepatitis (A, B or C), people at risk of drug overdose, veterans, people who were smokers, and people in prison. Studies with adults and/or youth were included.

A systematic search using six databases (CINAHL, SocINDEX, PsycINFO, MEDLINE, Scopus and Web of Knowledge) was conducted in August 2019 using search terms found in Table 1 and adapted for each of the searched databases. The search aimed to identify papers published in English from 1st January 2000. It included articles in peer reviewed journals and study protocols. A separate grey literature search was not performed. However, we decided that if a relevant report/grey literature source was identified as part of the reference list review of included papers (as detailed in ‘selection’ in Table 1), it would also be included in the final review. Only one such report was identified during this process, which was the Groundswell Report [27]. Synonyms of ‘peer intervention’ included terms such as ‘peer mentor’ and ‘peer counselling’ to accurately reflect terminology used in mental health and problem substance use services.

**Table 1:** Sample Search Terms

***Scopus***	
**Operator**	**Definition**
1. Title/Abstract/Keywords: Population	(homeless OR homelessness OR (homeless AND person*) OR (rough AND sleep*) OR housing OR (unstably AND housed) OR unsheltered)
2. Title/Abstract/Keywords: Population	((substance AND abuse*) OR (substance AND use) OR (drug* AND abuse*) OR (drug* AND addiction*) OR (drug* AND use) OR (alcohol*) OR (addict*))
3. Title/Abstract/Keywords: Intervention	((peer AND intervention*) OR (peer AND mentor*) OR (peer AND led) OR (peer AND support*) OR (lived AND experience*))
4. Boolean Operator	1 AND 2 AND 3
5. Language limit	English language
6. Time limit	2000–2019
7. Selection	Removal of duplicates followed by PRISMA guidelines of article sifting: title sift, abstract sift, full-text sift, review reference lists and articles citing
**MEDLINE**	
1. Title/Abstract/Keywords: Population	(homeless or homelessness or homeless persons or rough N2 sleeping or housing or unstably housed or unsheltered)
2. Subject Headings: Population	MH homelessness or homeless persons or houseless
3. Boolean Operator	1 OR 2
4. Title/Abstract/Keywords: Population	(substance abuse or substance use or drug abuse or drug addiction or drug use)
5. Subject Headings: Population	ZU drug abuse OR ZU substance abuse
6. Boolean Operator	4 OR 5
7. Title/Abstract/Keywords: Population	(alcoholism or alcohol dependence or alcohol abuse or alcoholic or alcohol addiction)
8. Subject Headings: Population	ZU alcohol abuse OR ZU alcoholism
9. Boolean Operator	7 OR 8
10. Boolean Operator	6 OR 9
11. Title/Abstract/Keywords: Intervention	(peer intervention* or peer mentor* or peer-led or peer support* or lived experience*)
12. Subject Headings: Intervention	(ZU “peer advice”) or (ZU “peer assisted programs”) or (ZU “peer assisted study”) or (ZU “peer case managers”) or (ZU “peer coach”) or (ZU “peer counseling program”) or (ZU “peer counselling”)
13. Boolean Operator	11 OR 12
14. Boolean Operator	3 AND 10 AND 13
15. Language limit	English language
16. Time limit	2000–2019
17. Selection	Removal of duplicates followed by PRISMA guidelines of article sifting: title sift, abstract sift, full-text sift, review reference lists and articles citing

PsychINFO via EBSCOHOST interface (83 papers); CINAHL Via EBSCOHOST interface (60 papers); Web of Science (1072 papers); MEDLINE via EBSCOHOST interface using all databases (70 papers); Scopus (340 papers); SocINDEX Via EBSCOHOST interface (29 papers); search conducted 22/08/19

**Fig. 1:**
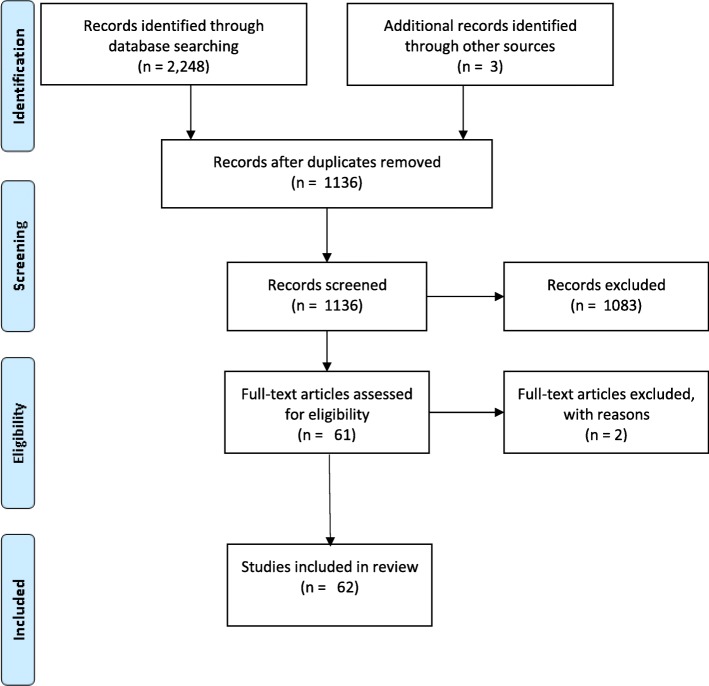
PRISMA 2009 Flow Diagram

We utilised a systematic approach to the literature search in two major stages. The first stage involved the first author (JM) screening titles and abstracts against the defined inclusion criteria to identify relevant studies to be reviewed in full. The search identified 2248 papers published in English, after the year 2000. This initial high volume is due to searching the full text of papers as well as title, abstract and key words, performed to minimise the risk of not capturing relevant data. After de-duplication 1136 papers remained for title/abstract scanning and 61 were deemed relevant. The second stage consisted of retrieving the full-text papers of the selected studies. JM documented study exclusions and reasons for exclusion at this stage. This process was also conducted in conjunction with another assessor (RF) who examined 10% of the included studies to ensure reliability. Three more papers were identified via references making a total of 64. Detailed information of this process is shown in Fig. 1 using a PRISMA flowchart [28]. After a close reading of the full texts, two articles were excluded due to lack of relevance (not focusing/mentioning substance use [29]); and not focusing/mentioning homelessness [30]) leaving 62 articles to be included in final synthesis. Again, to ensure reliability, the second assessor (RF) confirmed the exclusion of those papers. The final data extraction table was reviewed separately by all authors and there was consensus on the inclusion of the final included papers.

### Quality appraisal and study details

Quality assessment is not typically used as a study inclusion criterion in state of the art reviews [25]. Instead, papers are included based on their relevance. Methodological assessment may, however, be conducted to increase the transparency of the synthesis and the interpretability of the findings. We have thus assessed sample sizes, data collection methods, and perceived limitations of each included paper, although we did not use a formal quality assessment tool. The 62 included studies consisted of one grey literature report [27], 49 primary studies ([31–79]), including quantitative, qualitative, mixed methods and feasibility studies, three study protocols ([80–82]), four reviews ([26, 83–85]), three commentaries ([86–88]), and two case studies ([89, 90]). Sample sizes in the 52 primary studies ranged from *n* = 4 to *n* = 948. One study [67] had a sample of *n* = 268 but additionally looked at administrative data of other clients across the country (USA, *n* = 30,977). Data collection methods included semi-structured and unstructured interviews with peers, clients and stakeholders; ethnographic observations; repeated measures using standardised questionnaires; surveys and analysis of patient administrative data; and case notes.

Studies ranged in terms of their generalisability. The vast majority of the included papers were conducted in North America (USA *n* = 31 and Canada *n* = 16; with the other three countries being the UK, Portugal and France), and most of the primary studies were conducted at specific locations known to have high rates of socio-economic and health inequalities (e.g. the Downtown Eastside area of Vancouver was the setting for nine studies). These locations may have unique characteristics making findings less transferrable to other settings. Some limitations acknowledged by the primary study authors themselves included lack of blinding in trials (e.g. [66]), and small, self-selected sample sizes (e.g. [65] with 10 participants and [59] with 5 participants).

### Data analysis

Study characteristics, including setting, participant characteristics and methods, were entered into an Excel spreadsheet. A thematic analysis approach, led by JM and supported by HC, RF and TP, was used to compare the papers and provide a synthesis of the key points. We used Boyatiz’s [91] definition of a thematic analysis as ‘*involving the encoding of qualitative information through the assignment of manifest and/or latent categorisations’ [p. 7]*, with the purpose of ensuring meaningful data reduction. The process of the analysis was guided by Braun and Clarke’s [92] six-phase framework for conducting a thematic analysis, but adapted for the purposes of the state of the art review which includes both quantitative and qualitative data. These adapted steps were: 1) becoming familiar with the data (all 62 papers were read in full and details of the studies including quotations were extracted into an Excel spreadsheet; the data extraction table was read repeatedly); 2) searching for themes; 3) reviewing themes; 4) defining themes; and 5) writing up. Braun and Clarke’s [92] second step - generating initial codes - was conducted when first starting to populate the spreadsheet thus steps one and two were performed concurrently.

## Results

Included papers were published between 2001 and 2019 and there was a clear increase in published material since 2017 (31/62 papers were published between 2017 and 2019), with 15 published in 2019 alone (January–August). This suggests that there is a fast growing body of research on peer support, in tandem with an increase in the use of peer support in practice at the intersection of homelessness and problem substance use.

### Overview of included papers: primary topics of interest

The studies included in the review were diverse in terms of their primary focus/themes of interest (Table 2). They ranged from interventions targeting specific populations, for example peer support with individuals with HIV ([45, 60, 63, 71, 75]), or criminal justice involved/experienced individuals ([39, 58, 65, 73, 76, 82]), to focusing on specific harm reduction interventions or practices, for example needle exchange programmes ([31, 68, 70]) or safe consumption sites ([32, 56, 57, 78, 86]). The largest number (*n* = 15) focused specifically on peer interventions with individuals experiencing homelessness. These papers were methodologically diverse and included a systematic review, qualitative, quantitative and mixed methods studies, and a grey literature report.

**Table 2:** Overview of included papers (*n* = 62^a^)

Theme	Number of papers	Papers
Harm reduction (including needle/syringe exchange; safe/supervised injection consumption sites; naloxone training/distribution)	13	Ashford, Curtis and Brown (2018); Hayashi et al. (2010); Dechman (2015); Bardwell, Boyd, Kerr & McNeil (2018); Jozaghi & Reid (2014); Kennedy et al. (2019); Taylor H et al. (2019); Bardwell, Collins et al. (2017); Wright et al. (2006); Mitchell et al. (2017); Bardwell, Flemming, Collins et al. (2019); Poland et al. (2002); Collins et al. (2019)
Homelessness	15	Barker and Maguire (2017); Barker, Maguire, Bishop & Stopa (2018a); Barker, Maguire, Bishop & Stopa (2018b); Groundswell report Finlayson et al. (2016); Stewart et al. (2009); Parkes et al. (2019); Hunter & Power (2002); Pakhale, Kaur, Charron et al. (2018); Goldade et al. (2012); Charron et al. (2018); Pakhale, Kaur, Florence et al. (2016); Bardwell, Collins et al. (2017); Bean, Shafer & Glennon (2013); Crisanti et al. (2017); Tsai and Rosenheck (2012)
Abstinence-based programmes including AA/12 Step; relapse; and recovery	12	Blondell et al. (2001); Rayburn & Wright (2010); Boisvert et al. (2008); Tracy et al. (2012); Tracy, Guzman & Burton (2014); Tracy and Wallace (2016); Rosenblum et al. (2005); Ashford, Curtis and Brown (2018); Chapman et al. (2018); Ashford et al. (2019); Eddie et al. (2019); Davidson et al. (2010)
Smoking cessation	4	Pakhale, Kaur, Charron et al. (2018); Goldade et al. (2012); Charron et al. (2018); Pakhale, Kaur, Florence et al. (2016)
Physical health including: Tuberculosis, Hepatitis; and HIV	12	Croft, Hayward & Story (2013); Hirsch-Moverman et al. (2013); Deering et al. (2009); Nyamathi et al. (2001); Weeks et al. (2006); Swendeman et al. (2019); Latkin et al. (2003); Taylor J et al. (2019); Nyamathi et al. (2015); Tookey et al. (2018); Stagg et al. (2019); MacLellan et al. (2017)
Sub-populations including: veterans; people who have been in prison and criminal justice experienced individuals	11	Tsai and Rosenheck (2012); Ellison et al. (2016); McCarthy et al. (2018); Simmons et al. (2017); Resnick and Rosenheck (2008); Hebert et al. (2008); Krawczyk et al. (2019); Nyamathi et al. (2015); Lennox et al. (2017); Gonzalez et al. (2019); Bellamy et al. (2019)
Other	2 (history of development of peer support; 3 separate studies commentary)	Power (2002); Gardien and Laval (2019)

^a^62 papers; some were focusing on more than one major theme

The systematic review examined the effectiveness of intentional peer support (IPS) as an intervention with people who were homeless (including people who were street-dwelling and those within services [26]), termed ‘intentional’ because it is fostered and developed by professional organisations and can include either mentorship support or mutual support. There were two other papers looking specifically at IPS - a qualitative study looking into the experiences of peer providers and recipients and their opinion of what makes IPS effective in homelessness recovery [34]; and a mixed methods Q-sort study investigating the opinions of experts on what makes peer support effective with people who are homeless [35]. The remaining studies included: a quantitative pilot intervention of peer-led support for homeless youth [79]; a feasibility study of involving vendors of *The Big Issue* (a magazine sold by individuals who are homeless or at risk of homelessness that provides them with opportunities to earn an income) to become peer educators for people who are homeless to reduce drug related harms [55]; a study protocol for peer-led intervention for people who are experiencing homelessness and problem substance use [81]; a report from an independent evaluation of the Homeless Health Peer Advocacy Service programme on the impact on client’s health, cost, and impact on the peers themselves [27]; a commentary on the need for more safe/supervised consumption sites and other overdose prevention interventions across a range of housing sites to minimise overdose risk [83]; a longitudinal evaluation of a Housing First peer support model [36]; an evaluation of the effects of peer delivered permanent supportive housing on health and mental health of an ethnically diverse population [51]; an evaluation of a group intensive peer support model of case management in a supported housing programme for homeless veterans [67], and four studies relating to smoking cessation peer interventions in poly-substance using individuals experiencing homelessness ([47, 48, 54, 80]).

**Fig. 2:**
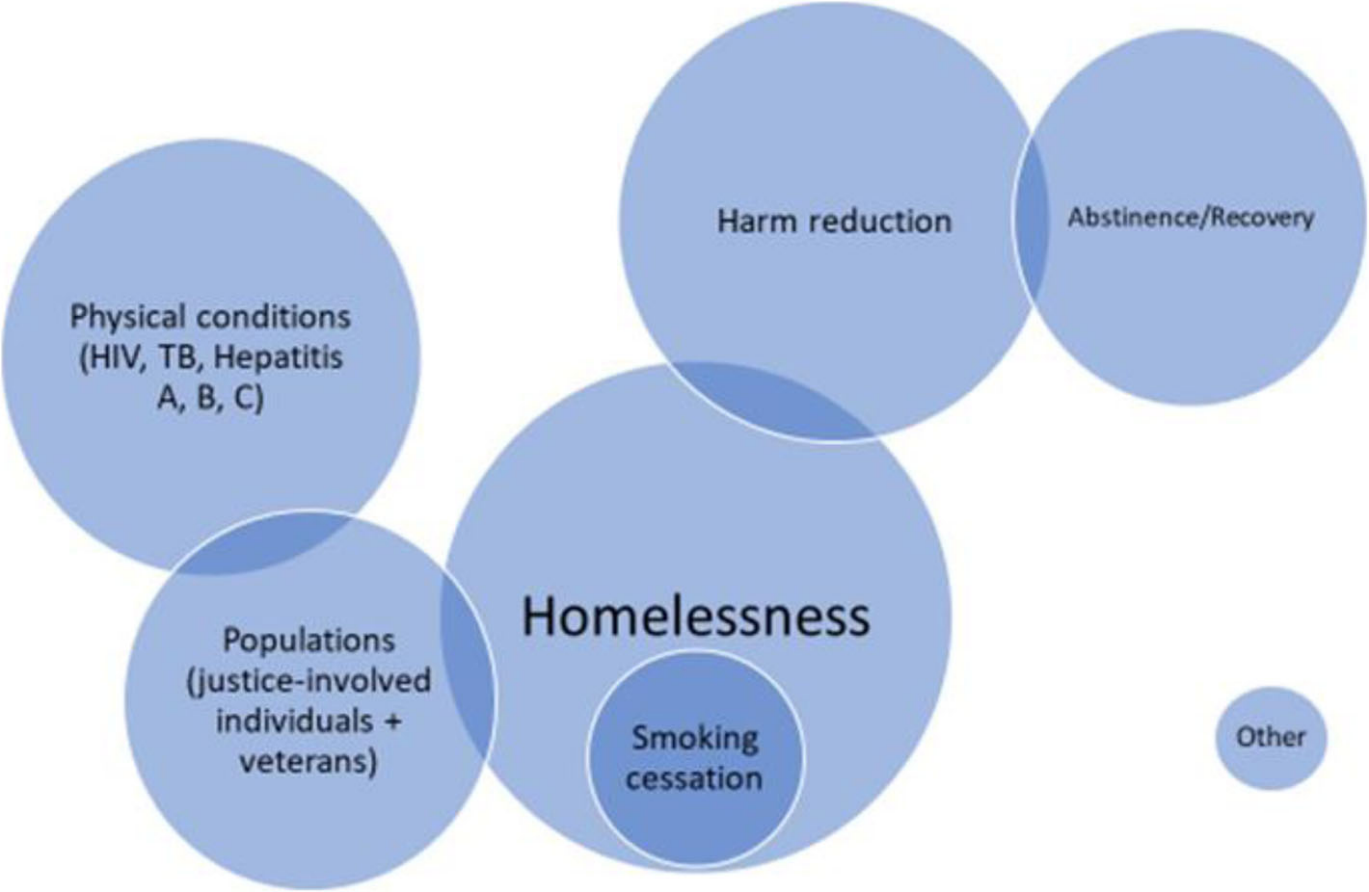
Venn diagram of key themes

There was some overlap between the key themes in the included papers, in particular all of the smoking cessation papers concentrated on providing peer support to individuals who were homeless. There was also overlap between the themes of justice involved individuals (including people in prison and individuals on parole) and the themes of physical health (including TB, HIV and Hepatitis (A, B and C)). Figure 2 displays a visual representation of the key themes using a Venn diagram. The sizes of circles correspond to the volume of papers with each key theme.

It is important to note that despite the fact that many studies on peer support have been published, very few explicitly focus on the intersection of homelessness and problem substance use. Some studies only mention homelessness or problem substance use briefly, or imply it. This was especially evident in papers discussing prison re-entry as people in prisons are at a high risk of homelessness upon release ([93, 94]), however homelessness is not explicit. Additionally, some papers included participants with problem substance use but did not specifically recruit them for this reason, nor did they focus on substance use (e.g. ([26, 34, 35]). Explicit focus on all three aspects of our chosen topic (peer support, homelessness and problem substance use) was clear in only 23 of the 62 papers ([32, 36, 38, 43, 44, 46, 48, 50, 51, 53–55, 59, 61, 62, 64, 67, 71, 72, 79–81, 89]).

Included papers varied greatly in terms of their inclusion of populations of interest and this was especially notable in terms of people with lived experience of homelessness. Studies ranged from having as few as 3.1% of their participants identified as ‘homeless’ or ‘unstably housed’ [56], to 100% of participants being homeless. Most studies which did not have an explicit focus on homelessness included samples that had approximately 30–50% of homeless participants (e.g. 30% in [42], 40% in [77] and 49% in [60]). Additional file1 shows the study characteristics in terms of participants and procedures. Data were also extracted on definitions of ‘peer’ and ‘homelessness’, peer characteristics, the types of peer involvement, and whether their involvement was voluntary or paid.

### Themes

#### Effectiveness of peer support/outcomes

Most of the included studies (*n* = 40) investigated the outcomes of peer support. Of those, 25 looked at quantitative outcomes of peer-run or peer-involved interventions, often without making any specific comments regarding the peers or their role, and only concentrating on outcomes of interest such as vaccine uptake or reductions in substance use. Fifteen looked specifically at the role of peers and factors which made them and their roles successful/effective using qualitative methods.

All of the studies reported some positive outcomes of peer-led/peer-staffed interventions. Barker and Maguire’s [26] systematic review of peer interventions with individuals who are homeless, which included 10 studies, found an overall reduction in harm related to drug and/or alcohol use. Half of the included studies reported reductions in drug and alcohol use and relapse rates, with two studies finding statistically non-significant changes related to problem substance use, specifically the amount of money spent on drugs or alcohol [36], and the number of days using drugs or alcohol [40]. Additionally, three studies reported improvements on homelessness, including decreases in the number of days spent homeless, a reduced return to homelessness, and reports of an overall improvement in housing environment ([36, 38, 95]), but one study found no improvements [96]. Tracy and Wallace’s [85] systematic review assessing the use of peer support groups in the treatment of problem substance use found that those who participated in any type of treatment, including peer support groups, showed higher than expected rates of abstinence, significant reductions in relapse rates, and higher levels of satisfaction with treatment. However, it is important to note that this review did not focus on homelessness, and of the included ten studies only one explicitly discussed homelessness [38]. Boisvert [38] reported that the return to homelessness was dramatically reduced by assisting participants in managing their recovery from problem substance use.

In the quantitative studies, Bean and colleagues [36] evaluated Project H3 (Homes, Health, Hope), which involved the provision of housing, harm reduction and peer support using a Housing First approach. This is an approach to ending homelessness that focuses on providing immediate, permanent, low-barrier, non-abstinence-based supportive housing for individuals with lived experience of homelessness [97]). The authors reported that 98% of participants remained in housing after 12 months. They also observed statistically significant changes in participant reports of substance use, quality of life, and use of primary care physicians, between baseline and six months. Similarly, Blondell and colleagues [37], in their study of those with lived experience of problem alcohol use helping hospitalised patients with alcohol problems, observed significantly higher rates of abstinence from alcohol from discharge in the peer support group (59%) than in the control (34%) and brief intervention (44%) groups. Moreover, those in the peer group had significantly higher rates of initiation of treatment/self-help (49%) than those in the control (9%) and brief intervention (15%) groups [37].

Pakhale and colleagues [48] conducted a mixed methods feasibility study of a peer intervention for smoking cessation for people who were homeless and using drugs called the Participatory Research in Ottawa, Management and Point-of-Care of Tobacco (PROMPT) study. Their quantitative data revealed that the six-month follow-up rate was 43%, with a significant reduction in mean daily cigarette use from baseline (20.5 cigarettes per day (CPD) vs 9.3 CPD). The authors also found a considerable reduction in self-reported illicit drug use, including a reduction in the use of opioids such as heroin, fentanyl and oxycodone. The study findings also described psycho-socioeconomic benefits, such as improved physical health, return to work and greater community engagement.

Ashford and colleagues [31] evaluated a hybrid recovery community organisation providing peer recovery support services, as well as peer-based harm reduction via a syringe exchange programme, and found that a total of 895 peer engagements with 417 individual participants occurred in the study period. This suggested that there was a need for a peer support initiative of this kind and that the intervention was having a wide reach. The authors also found that those who were homeless were the least likely to engage with the peers multiple times, and that bisexual people and Latino people experienced difficulties in engaging with the project [31].

On the other hand, some studies only reported modest positive outcomes, or no differences from standard treatment/other existing interventions. For example, Nyamathi and colleagues [63] examined the six-month impact of three cognitive behavioural HIV risk reduction programmes (peer mentored vs nurse case-managed vs standard care HIV risk reduction programme) on behavioural factors (problem substance use and sexual risk behaviours) and cognitive and psychological resources of women residing in emergency or dry shelters and their intimate partners. The authors found modest to marked improvements among the participants for all three groups, and significant changes over time were found for all outcomes apart from self-esteem (which increased significantly in the nurse-case managed group only). Depression lessened in the peer and standard care groups but not in the nurse case-managed group; and hostility decreased significantly in the peer group only. Similarly, Hirsch-Moverman and colleagues [69], in their randomised controlled trial regarding a peer-based intervention’s impact on adherence to treatment for latent TB infection, found a non-significant difference between experimental and control groups: 61% in intervention vs 57% in control groups completed treatment. They also found that being currently homeless and currently using alcohol were significant predictors of not completing treatment. Tsai and Rosenheck [67] evaluated a Group Intensive Peer Support (GIPS) model of case management in a supported housing programme for veterans who were homeless. They reported that GIPS can be as effective as (but not more than) an intensive community management programme. They also reported that the only significant difference between it and the intensive community management programme was a greater increase in social quality of life scores over the course of six months [67].

In terms of qualitative studies, Jozaghi and Reid [56] examined the role of peers who volunteered at safe injection/needle distribution sites in transforming the lives of people who inject drugs. They found housing to be a common theme, with peers supporting people to find housing either for the night or more secure, social housing. The authors note the importance of activism: peers fighting to secure extra funding and support for safe injecting and other harm reduction facilities in Vancouver’s Downtown Eastside, and to change perceptions of people who use drugs. The peer workers who were interviewed believed that there had been a reduction in risky drug use behavior, at least in part because of their role in the distribution of injecting supplies. This was starkly contrasted with the previous situation, where it was difficult to access needles and people were forced to share. The peer workers in this study held a belief that supervised injection facilities and needle exchange facilities reduced the risk of overdose death and reported continually being on the lookout for signs of overdose. The peer workers also had a role in providing peer education, which resulted, albeit implicitly, in reduced mortality and reduced rates of HIV. Lastly, the peers also contributed to changing the culture in services: for example through informing people about diseases, recommending the use of harm reduction mouth pieces, and taking extra syringes. This advice enabled service users to establish safer injection habits. Jozaghi and Reid [56] also reported that the peer workers discussed how the relationships that were built promoted access to drug treatment services as well as how a sense of community was fostered: *‘more than a place to do a fix’ (p.17)*, *‘almost like a family’* (p.18). Moreover, the authors reported positive changes to the peers’ behaviour, with some reporting that involvement in the project helped them to abstain from drug use [56].

MacLellan and colleagues [59] explored how peer advocates with experience of homelessness and problem alcohol and drug use made and sustained relationships with their Hepatitis C positive clients with experience of homelessness and injecting drug use. The authors reported three main ‘techniques’ used by the peers to achieve ‘connectedness’ through establishing a positive therapeutic alliance with clients: rapport; self-disclosure; and shared group membership with health services. Peers talked about establishing connections with their clients using respect, reciprocity, and friendship: *‘I think they see me more on their level sort of thing, err, not a friend exactly but someone who is on their team sort of thing’ (*p.4*)*. Peers also talked about their ability to enjoy membership of both client and staff groups, and being able to act as a bridge to a client’s successful engagement with services.

In the qualitative component of the PROMPT smoking cessation study, Pakhale et al. [48] interviewed 80 service users and four peers and identified a number of themes regarding the role of the peers and their impact on participant outcomes. Many of the participants believed that their progress was due to the support they received from the project’s peer researchers. The peer researchers’ leadership and coordination of monthly follow-ups, outreach and project-related workshops created a sense of community and connection that many participants reported finding helpful in managing, and in a sizeable number of cases reducing or altogether stopping their concurrent problem substance use (19%), and in helping them to reduce or quit smoking. This programme focused on tobacco dependence, was easily accessible in the community, was led by community peers with lived experience, and was feasible to implement. It therefore had the potential to support positive life changes. The authors concluded that the PROMPT’s patient engagement model was an effective harm-reduction strategy leading to considerable psycho-socio-economic benefits such as better health (weight gain, improved breathing and physical exercise, enrolments in school and drug treatment) and social outcomes (returning to the work force, improved housing, reunification with family, greater community engagement) and thus could improve the lifes of marginalised at-risk populations worldwide [48].

In another mixed methods study, Barker et al. [35] utilised Q-sort methodology to examine expert, both peer and professional, opinions on what makes effective peer support with those who are homeless. The peers generally agreed that confidentiality and training were important, but that training was not vital for effective peer support. Professionals felt that it was important that peers were able to be positive role models, providing emotional social support and creating a bridge between clients and professionals, but believed that successful peer support does not depend on peers knowing specific services, or even having genuine motivations to provide support. Both peers and professionals also felt that in order to be effective, peers needed to be adaptable, committed, provide emotional social support, and listen empathically to clients. Both groups also felt that the peers needed support from supervision and from other peers in order to positively influence client outcomes. Participants acknowledged and valued the uniqueness of peers, their difference from professionals, and their ability to develop strong, trusting, experience-based relationships with clients. Both groups thought that peers were especially effective in being able to approach clients on an equal level, and that they had a distinct ability to understand the client perspective based on their shared experiences. Lastly, both professionals and peers agreed that peers were able to develop trust with clients, and this was viewed as a key ingredient of effective peer support [35].

#### Challenges for peers

Eleven of the included papers looked at the challenges that those in peer support roles can face ([34, 43, 47, 55, 57, 59, 65, 70, 88–90]). Five key themes emerged: vulnerability; authenticity; boundaries; stigma; and a lack of recognition of value of peers and these are now discussed.

### Vulnerability

Often vulnerability related to a peer worker’s own recovery and there was an implication that working with people who have experienced problem substance use could lead to relapse ([34, 90]). In Barker’s et al. study [34] peer supporters discussed the need to know themselves, control their emotions and identify triggers to maintain their recovery. They expressed the need to be secure in their own recovery to be able to cope with any situation that occurs: ‘*Knowing your limitations for yourself … it’s just knowing what you can do and what you can’t do. Yeah, it’s just them being aware of their own triggers … it’s a hard one because you … you never … you don’t know who you’re going to meet*’ ([34] p. 222). Moreover Barker et al. [34] identified the recovery of peers as a potential obstacle, for example, relating to the minimal length of time in recovery from drugs and/or alcohol which may be imposed by a prospective employer and which may be a barrier to those assuming a peer role.

Others suggested that challenges for recovery can also be seen in terms of the workplace and that adjustments may have to be made to allow for flexible working for peers so that they can maintain their own recovery ([34, 90]). For example, Chapman et al. [90] sought to identify and assess best practices in peer provider workforce development and employment and conducted 194 interviews with professionals across four US states. These expert opinions concluded that peers may require workplace accommodations to maintain their recovery and that allowing this should be seen as best practice. The authors reported that some employers responded to this need for accommodations through leave of absence policies and the provision of one-to-one or group supervision for peers, allowing opportunities for checking in on one’s recovery, additional training, and client updates. However, several interviewees working in human resources reported that peer support staff required no more accommodations than any other staff [90].

Another form for vulnerability related to the amount of responsibility that peers had to assume in their roles and how overwhelming this could be. Dechman [70] interviewed peer workers in a needle exchange programme about the challenges they experienced. Peers stated that they may be the only connection many service users have to any form of medical advice, and that they often found themselves caught between the very limited forms of harm reduction assigned to their official role and the very real life threatening harms associated with infection, drug use, overdose and marginality (‘*I might be their only option’* (p.496); *‘A lot of people can’t inject themselves so I’ll inject them. I don’t want to but I want them to be safe*’ (p. 497)). Similarly, Wright and colleagues [43], in their qualitative exploration of peer provision of take home naloxone (THN), saw a clear theme of willingness to administer THN in an emergency situation but many peers worried about being charged with unintentionally causing death. Charron et al. [47] assessed community researcher training and experience in the PROMPT project (the same intervention as [48]) and noted that peers felt accountable as project leaders, as illustrated in this quotation from one of the peers: *‘Service providers can just go back to the office but as a peer you can’t hide from your own community’* ([47], p.7).

Lastly, there were vulnerabilities associated with the lack of support for peer workers. For example, Kennedy et al. [57] explored the role of peer workers at overdose prevention sites in Vancouver and noted that alongside clear benefits to the peer workers there were also challenges, including grief, trauma and a lack of support services. Many of the interviewed peer workers revealed that they had lost at least one friend or family member to overdose death, and also routinely encountered overdose events while working at overdose prevention sites and in the broader community. This contributed to experiences of considerable trauma and grief due to the emotional toll of the overdose epidemic and a lack of adequate support: ‘*When you see your friends go down or you come across your friends and they’re dead, like it’s – it really really gets to you after a while … […] … At the end of the day, we’re all hurting and we all lean on each other*’ ([57], p., 65). Many of the interviewed peer workers discussed how these feelings contributed to burnout in regards to their roles as peer workers, resulting in feelings of emotional exhaustion and disconnection from their work. In some cases this had led to people taking a reduction in the number of shifts worked, or termination of their peer positions altogether: ‘*I’ve spent so many years sitting in rooms watching people like this … It’s really hard for my post-traumatic stress, sitting there watching that go on… so that’s why I haven’t been putting in as many hours there*’ [57], p., 65.

Kennedy et al. [57] reported that as well as experiencing grief and trauma, peer workers were also not treated the same as ‘standard workers’, especially in relation to the availaibilty of support services for the range of difficult emotions and experiences commonly encountered in their roles. Most of the peer workers were not offered the employee benefits and support services (e.g., health benefits, counselling, stress leave) typically afforded to salaried non-peer employees in similar positions at other local organisations. Interviewees highlighted the need for interventions to address gaps in social and emotional support provision for peer workers experiencing trauma, grief and other adverse psychosocial responses resulting from their peer roles: ‘*I think that’s one thing they should think about setting up, is some place to go to talk about this, right? Because a lot of people just want to, you know, explain their feelings and stuff, which is – you know, there’s no better therapy than talking, right?*’ [57], p., 66.

### Authenticity

Issues of retaining authenticity were identified in four of the included papers. Gardien and Laval [88], in their analysis of the institutionalisation process of the role of peer support workers in France, posed the question of whether peers should be given ad hoc training or whether their lived experience should be seen as sufficient. They suggested that this leads to a further question: *‘wouldn’t training them be to risk formatting their experience according to professional precepts*?’ (p.76). In Hunter and Power’s [55] feasibility study of a peer education intervention by *Big Issue* vendors, this dilemma was identified by peers who believed that life experience should be seen as a qualification for delivering a peer intervention.

Not only was the question of training versus lived experience in relation to authenticity of peer workers discussed in the papers, but lived experience versus recovery was also seen as problematic in relation to maintaining peer identity and subsequent authenticity. For example, Tookey and colleagues [89] looked at two case studies of the transitions, facilitators and challenges of moving from being a client to a peer worker, for drug using former clients with hepatitis. They found that peers can feel like they are no longer authentic, and are no longer ‘true peers’, the longer they are in recovery, or the more positive their own lives become: *‘I feel like the more clean time I have and the more my life has changed and progressed on from that [client stage of life], I feel like my buy-in is becoming...less’ (p.8)*. Similarly, Barker et al. [34] identified that peers help in four main ways: being role models, breaking boundaries, providing individualised treatment, and offering social support. They also noted, however, that some participants voiced discomfort for being seen as a role model, or appearing ‘different’ from, or ‘better’ than their clients. Another issue regarding authenticity that is worth contemplating relates to the conditions of peer employment: formalisation of peer engagement comes with some boundaries and ‘regulation’ (e.g. [98]). Engagement is more than just ‘peers exchanging experiences’, as they might do in a setting where there is no professsional/service environment or input. It would therefore be helpful if discussions around authenticity were undertaken with peers prior to them starting their roles.

### Boundaries

This theme was apparent in three papers ([59, 89, 90]). Tookey et al. [89] noted that peers discussed experiencing shifts in relationships with community members and friends and needing to establish boundaries between themselves and their clients. In some cases they discussed transitions between themselves and their former communities, which also involved making personal changes outside of work, such as someone deciding to move to a ‘better’ area/housing. MacLellan et al. [59] noted that despite peers uniformly commenting on the need to maintain rapport and build friendships with their clients, they also countered this via a narrative of maintaining boundaries, and of selective self-disclosure: *‘so you’ve got to share a bit but not too much*’ (p.4). Similarly, Chapman et al. [90] commented on the challenges peer workers, who are themselves in recovery and may experience relapse, faced in setting up and addressing boundaries. This required a skillful negotiation to then acquire a balance of empathy and self-disclosure while maintaining professional boundaries.

### Stigma

Stigma was explicit in two papers ([47, 90]). Chapman et al. [90] concluded that stigma, and the perception of stigma, regarding employing workers with lived experience of problem substance use/homelessness remained a problem in the workplace and included labelling, stereotyping, and experienced or internalised discrimination. Problems with acceptance and stigma were reportedly more common during interactions with non-peer staff in clinical and forensic settings in particular. Chapman et al. [90] noted that some of the non–peer-run organisations required front line staff and those in leadership roles to attend training on the peer provider role in order to address issues of stigma before introducing peers. Charron et al. [47] found that stigma was also apparent within the peer community itself. They reported that peers did not expect as many of the study participants to reduce or quit tobacco use (79% reported reducing tobacco, 9% reported quitting, 19% reduced poly substance use), stating *‘when people stopped coming I thought they might be out partying when in fact some were getting jobs, going to rehab, or in the hospital getting better. At times we stigmatize our own community and ourselves’* (p.7).

### Recognising the value of peers

Seven studies described the importance of recognising the value of peers ([34, 47, 55, 57, 65, 88, 90]). Gonzalez et al. [65] looked at qualitative outcomes of peer re-entry specialists on housing attainment, mental health and substance use. Peers in that study were assigned to clients based on gender and native language but the authors found that peers would have preferred for the assignment to be based on their individual strengths and lived experiences: ‘[*one of our peers] is experienced with the alcohol recovery and drug recovery world. For a lot of her [clients], she’s been amazing in getting them into recovery centers and working with them and I think that’s because she knows so much […] So yeah I think that lived experience might be a factor; which is good I think because then we’re matched up with people that were better able to help*’ (p.1867). Peers suggested that lived experience ‘*makes someone an amazing person because they have lived it overcame it and now they’re giving back with it*’ ([65], p.1868). However, several peers perceived their role to be undervalued in their work environment: *‘I do think that peer title holds us back in some areas. And I think someone needs to look how beneficial we are because we’re doing progress notes, we’re doing tons of paperwork. Plus, we’re [compiling] resources, plus we’re meeting with the client… we do so much more than so many. And it’s overlooked’ (*p.1868*)*. Similarly, Charron et al. [47] found challenging power issues between academic and peer researchers: *‘I felt that since I did not have as much education as others my contribution was not as valued’* (p.6). Gardien and Laval [88] described nurses who viewed the new peer support workers as a major threat, creating ‘an outcry’. Similar to the issue of stigma coming from within the community, Hunter and Power [55] also highlighted the potential of lack of respect for peers not only from the wider workforce but from within the peer communities themselves, and a sceptism voiced by the *Big Issue* vendors that other drug users will not take the advice of peers, despite their ‘peer status’.

Five papers explicitly discussed issues of adequate compensation for the work of peers and opportunities for professional development ([34, 55, 57, 88, 90]). Gardien and Laval [88] highlighted a lack of clarity regarding the peer role and disagreement regarding the various terms, questioning whether a new occupation had been created (peer support worker) or whether this is just a new name for ‘volunteers’. In France peer workers only receive minimal pay and have no prospect of advancement in a professional career. Their status as an employee is therefore legitimised but their employment conditions are poor [88]. Similarly, in a US study, Chapman and colleagues [90] note several key themes that have implications for the growth of peer provider employment nationwide: roles and job descriptions in various employment settings; training and certification approaches; billing and reimbursement for peer providers; and workforce and career development. They note that peer providers are often low-wage workers with limited opportunities for career growth and may require workplace accommodations to maintain their recovery. Kennedy et al. [57] also highlighted that peers are underpaid, not sufficiently valued, with tokenistic involvement. Hunter and Power [55] emphasise that having a financial incentive was important for participation: *Big Issue* vendor involvement in peer intervention would not be possible unless lost vending costs were reimbursed. Barker et al. [34] recounted that some of the peers with volunteer status believed that they would feel more valued if they got paid, yet others believed that the lack of payment was what made them different to professionals. This issue of compensation is a complex one. It is of course essential to reward and avoid exploitation, however it is important to note that the circumstances of, and resources for, peer engagement vary significantly (e.g. [23]). In addition, the rationale for volunteering can be very strong, especially in relation to development of needed skills and experience with additional support structures, and the presence of social welfare restrictions regarding an individual transitioning back into paid employment (e.g .[83]). Full information and transparency is therefore vital for peer workers on their specific ‘terms of engagement’.

## Discussion

To date, no systematic review focusing specifically on the intersection of homelessness and substance use has been published. A number of reviews are available regarding peer support for people who are homeless (e.g. [24]) as well as for people with substance use problems (e.g. [83]). However, it is important to acknowledge that despite high rates of substance use in the homeless population, not every individual who is homeless uses substances, and not every individual who is experiencing problem substance use is homeless. This is why it is particularly important to look at this specific group of indivduals who have arguably some of the most complex needs yet require significant amounts of support, in part due to the extensive barriers they encounter when trying to access help. Relatedly, it is important to acknowledge that the provision of peer support is also likely to be more complex, nuanced, and unique than peer support focused on responding to one of these challenges in isolation.

This state of the art review included 62 studies published between 2001 and 2019. A clear increase in published material since 2017 has been observed, with 15 of the included studies being published in 2019 alone (January–August). Peer support is increasingly gaining in credibility and popularity, alongside strategic policy acknowledgement, as noted above [22], and the connections between the lived experiences of both homelessness and problem substance use is becoming more visible in social research. The included studies were very diverse in terms of their primary focus and themes of interest and ranged from interventions targeting specific populations to historical commentaries on the rise of peer support as an emerging profession. Despite the marked increase in publication of studies on peer support, it was very rare for these studies to explicitly focus on the intersection of homelessness and problem substance use. Only 23 of the 62 papers clearly focused on peer support, homelessness and substance use together, rather than merely including these groups of clients in their study samples. The included papers also varied considerably in terms of their inclusion of populations of interest, something particularly notable in terms of people with the lived experience of homelessness. This variation was unsurprising given it reflected the aims of the different standalone studies, with only a proportion of them focusing explicitly on homelessness. Studies ranged from having as little as 3.1% of their participants identified as ‘homeless’ or ‘unstably housed’ ([56]), to having 100% of participants being defined as homeless ([48, 54]).

Most of the included studies investigated the effectiveness of peer support and all studies reported at least some positive outcomes of peer-led/peer-staffed interventions, including seeing an overall reduction in substance use-related harm; reductions in drug and alcohol use (with some studies reporting abstinence); reductions in cigarette use and increased smoking cessation; improvements in homelessness status including housing retention; and psycho-socioeconomic benefits such as improved health, return to work, and greater community engagement leading to improvements in quality of life. A small proportion of the included studies reported modestly positive outcomes only, or no differences from standard treatment/other existing interventions.

These findings echo those from reviews of the effectiveness of peer support for those with substance use problems. For example, in a recent systematic review Eddie and colleagues [83] summarised the existing quantitative research on peer recovery support services and concluded that current findings tentatively speak to the potential of peer support across a number of substance use treatment settings. This is evidenced by positive findings on measures including: reduced substance use and relapse rates; improved relationships with treatment providers and social supports; increased treatment retention; and greater treatment satisfaction. However, the authors urge some caution when considering these findings, in light of many null findings to date. They also highlight significant methodological limitations in the existing literature, including: the inability to distinguish the effects of peer recovery support from other recovery support activities; the heterogeneous nature of these populations, inconsistency in the definitions of peer workers and recovery coaches; and the lack of any, or appropriate, comparison groups [83]. Similarly, findings to date regarding the effectiveness of peer support in mental health arena remain mixed [18].

Qualitative studies in our review emphasised that peers can have far reaching positive impacts on the lives of their client, and that their work displaying leadership and supporting the coordination of meetings, undertaking outreach activities, and project-related workshops, could help to create a sense of community and connection which could, in turn, lead to better management of concurrent problem substance use and other issues for the participants. Peers that were interviewed in these studies discussed relationships that promoted access to drug treatment services and a sense of community: *‘almost like a family’* ([56] p.18). Alongside the benefits of peer interventions for service users, peer interventions can also benefit the peer workers themselves and lead to changes in their own behaviour; for some the involvement in the projects helped them to abstain from their own drug use ([56]).

Despite the identified benefits, a substantial proportion of the included papers identified challenges that peer workers commonly face in these roles including: vulnerability; authenticity; boundaries; stigma; and having their involvement valued. It is clear from this review that most peers and professionals can now recognise the unique position of peers, including their ability to create a special type of rapport based on shared experience and lack of judgement, and their ability to gain trust. Many peers and professionals understand how valuable this is in engaging people with multiple social and health inequalities and connecting them to wider supports and services.

Some of our findings echo those from other reviews on peer support in other spheres, for example in mental health. Davidson and colleagues [17] reviewed the literature on the unique contributions of peer support and reported them to be: a) the instillation of hope through positive self-disclosure and demonstration that it is possible to re-gain control over one’s illness and move away from being a victim to being the hero of one’s own life journey ([97, 98]); b) exploration of new ways in negotiating day-to-day life, not only with the illness but also with having little income, being unstably housed, overcoming stigma, discrimination, and other trauma ([99, 100]); and c) the nature of the relationship between peer supporter and recipient, which is thought to be essential for the first two components to be effective. This relationship is characterised by trust, acceptance, understanding, and the use of empathy; and the ability to “read” a client based on having been in the same shoes he or she is in now [17]. These findings suggest some universal aspects and contributions of peer support, common across the various domains and services utilising it.

Despite this positive recognition of peer contributions there is still a lack of clarity regarding the peer supporter role and disagreement and confusion regarding the various terms being used to describe their work. While variation in interpretation may be appropriate and necessary, for different circumstances, and to enable innovation, there might be benefit in more consistency and clarity regarding these roles in both research and in practice, including in the terminology used. The peer support worker status as an employee in some settings can be legitimised but terms of employment are not always ideal, with lack of opportunities for professional development, and unfair and inadequate salaries, commonly highlighted. In some settings peer workers are engaged as unpaid volunteers which can be problematic and lead to feeling undervalued, as well as not providing career progression opportunities. In many of the included studies in this review there was no mention of pay and compensation for the peer workers which serves to highlight this problem. As noted earlier, however, this is a complex element of the provision of peer support, especially in relation to social welfare/security and the benefits system, and the desire that many people have to ‘give back’ and help those struggling with issues that they have also experienced.

### Implications for policy, practice and future research

Based on the synthesis of the key themes in this review, we now propose a set of guidelines for the sector to consider (Tables 3 and 4). These recommendations address both how to effectively and transparently present research involving peer support interventions for people with problem substance use and housing challenges, and how to embed peer support interventions in services in ways that do not create or exacerbate vulnerability and stress for those holding such roles.

**Table 3:** Proposed guidelines for dissemination of research involving peers

Proposed guidelines for dissemination of research involving peers	
Describe role	Clearly describe the role peers played throughout the project/intervention
Involvement	Provide details regarding the involvement of peers in: study design; intervention; delivery of services; data collection, analysis, and reporting
Outcomes	Provide detail on the effect of peer involvement on the outcomes of interest, and how the effect was achieved
Recognition	Provide detail on how peers are valued such as through the provision of training and development opportunities, and fair remuneration.

**Table 4:** Proposed guidelines for embedding peers in services

Proposed guidelines for embedding peers in services	
Role description	Clear description of role / job needed to prevent peers from assuming extra responsibilities beyond their contractual tasks, overworking and burnout.
Compensation	Transparency must be ensured in terms of compensation for the service provided so that peers can make informed choices regarding their terms of engagement. Recognition of the complexity regarding compensation and social welfare/security issues is needed. Low waged work should be challenged especially where peer roles are demanding and complex.
Support	Support services must be available so that peers can feel emotionally supported given the difficult nature of their roles.
Development	Training and development opportunities must be available to ensure career progression.
Value	Value and recognition of peer workers must be ensured. Peers should feel welcome and included in their workplace and by other colleagues.
Accommodations	Workplace accommodations should be in place as required by each individual.

When disseminating research involving peers there are key elements that need to be clearly presented. It would be helpful if authors provided information about the role of the peers, in terms of their involvement in the study design, the intervention, the delivery of services, data collection, analysis and reporting. There should be clear descriptions of the effect of peer involvement on the outcomes of interest, and how peers helped to achieve the outcomes. Lastly, transparency is required in reporting compensation/pay and condition that then enables peers to make informed employment/volunteering choices, or whether peers were provided with training and supervision opportunities.

Embedding peers in services has implications for research, policy and practice and these demand careful consideration. Peer workers commonly lack standard workplace benefits including access to support services, training opportunities, fair pay and conditions, and career progression. It is vital that peer workers are treated fairly and comparably to other employees and this includes: having a clear job role and description so that they are not overworked or forced to assume extra responsibilities beyond contractual tasks; being given adequate compensation for the service they provide; being given training and development opportunities with an ability to progress in their careers; providing support services given the difficult nature of these jobs; ensuring that peers feel valued and recognised and feel part of the workplace environment; and lastly, ensuring that there are workplace accommodations in place where needed. We offer this while acknowledging that implementation of these recommendations will necessarily be resource dependent and require cultural changes to take place to value the role of peer workers more highly. Environments that peer workers (and other support staff) encounter tend to be pressured and challenging in a range of ways, with both peer/non-peer staff at risk of overworking and subsequent stress. Most of the guidelines presented below can therefore also be applied to other staff working within support services at the intersection of homelessness and problem substance use. Moreover, it is important to acknowledge that some of the issues and concerns presented here have also been identified in reviews regarding peer support provision in mental health settings (e.g. [17]). This again suggests the significant challenges faced by peer supporter workers in their roles, irrespective of service type or context.

### Strengths and limitations

This state of the art review has captured the increasing involvement of peers in peer-led/peer-staffed interventions as well as in research projects, especially in the past two years, across both homelessness and substance use services/populations. Peer support at the intersection of homelessness and problem substance use is effective and provides benefits to those using services and to peers themselves. We have also highlighted a number of issues and challenges in conducting research with peers, as well as challenges that peers commonly face in their roles. The contribution of this review is novel in that it synthesises, for the first time, the common themes highlighted across the varied peer support literature, enabling conclusions to be drawn. The review has provided the opportunity to learn valuable lessons and use these to present a set of guidelines for policy, practice and research to address common and widespread challenges in implementing these roles.

Throughout the review, many steps were taken to enhance rigour: all stages of searching, screening, quality appraisal, data extraction, and analysis were checked for accuracy by at least two people. Issues with the quality and clarity of some of the included papers were noted. For example, in the quantitative studies peers were commonly described in a tokenistic way, without attempting to disentangle what the role actually involved in practice, and how peer involvement paid a specific contribution to the observed positive outcomes. Another identified issue was that many studies did not report whether peers were paid or not which is problematic and should be reported, given the implications for value and recognition of these roles.

The majority of studies (*n* = 47) were conducted in the USA and Canada which may limit transferability of the findings to other countries. This may be particularly evident when trying to compare the support and treatment options for those experiencing homelessness between the UK and North American contexts, given the substantial differences in systems for housing, healthcare (including substance use treatment), criminal justice system and welfare payments [101]. Indeed, the types of peer involvement practices differed within the US context itself ([90]). It would thus be helpful for this field if more work was conducted in other countries.

Finally, we acknowledge that while our paper takes a rigorous approach to systematic review and synthesis of a wide range of empirical studies and contexts it does not engage with theoretical explanations for the findings. This partly reflects the nature of the review and partly its aim and scope to synthesise the literature, distill important learning for policy, practice and research, and present these within new guidelines. We will be addressing this in a complementary paper that focuses on the role of peer workers in specifically preventing substance use-related harms.

## Conclusions

When peers support clients, the benefits are most keenly experienced by these clients, but are also experienced by services – both host and collaborating services, professionals and the peer workers themselves. The findings from this state of the art review reinforce the wisdom of the growing presence of peer-led interventions in a wide range of contexts. The review has also identified considerable challenges and risks for these roles and for those that undertake them. Based on the learning of benefits and challenges, we present a set of guidelines and recommendations for research, policy and practice to further develop this important area of work and ensure that peer workers and their contributions are valued, well supported and compensated.

## Supplementary information

**Additional file 1: Table S3.** Data extracted from included papers.

## Data Availability

All data generated or analysed during this study are included in this published article [and its supplementary information files].

## Abbreviations

AIDS: Acquired Immunodeficiency Syndrome
CPD: Cigarettes Per Day
GIPS: Group Intensive Peer Support
HIV: The Human Immunodeficiency Viruses
IPS: Intentional Peer Support
PROMPT: Participatory Research In Ottawa, Management And Point-Of-Care Of Tobacco
TB: Tuberculosis
THN: Take Home Naloxone
