# Supplementary material for: Provision of peer support at the intersection of homelessness and problem substance use services: a systematic ‘state of the art’ review
Source: BMC Public Health. 2020 May 7;20:641. doi: 10.1186/s12889-020-8407-4 (PMC7203893; doi:10.1186/s12889-020-8407-4)
Supplement: Supplementary file 1 — Additional file 1: Table S3. Data extracted from included papers. [file 12889_2020_8407_MOESM1_ESM.docx]

Additional file 1: Data extracted from included papers

| **Authors** | **Specific intervention (Y/N)** | **Design** | **Methods** | **Participants (n)** | **Results** | **Definition of peer** | **Definition of homelessness** | **Definition of substance use** | **Nature of peer involvement** | **Were peers paid?** |
| --- | --- | --- | --- | --- | --- | --- | --- | --- | --- | --- |
| **REVIEWS** | | | | | | | | | | |
| Barker and Maguire, 2017 (UK) | Yes – Intentional Peer Support (IPS) | Systematic Review of effectiveness of IPS for those experiencing homelessness | Systematic review searching databases (PsycInfo, Web of Science, MEDLINE and Cinahl) and grey literature. | 10 studies included. Baseline data from 1829 participants, complete data from 1341. The most common population was adults experiencing homelessness and dependent on substances (494 participants from 4 studies). | Overall reduction in harm related to addiction. Half the studies reported reduction in drug and alcohol use, and reducing relapse rates. 3 studies report improvements on homelessness - one found no improvements. | SAMSHA ‘individuals who have common life experiences with the people they are serving’ and 'have a unique capacity to help each other based on a shared affiliation and a deep understanding of particular experienes’. | The Housing act 1996: 'someone who lacks accommodation, cannot access accommodation, or resides in a vehicle or building which is unsuitable for occupation (Bennet et al, 2005, p.9). In this review, adults and young adults including streetdwelling and those engaged in services. | No | Varied across the 10 included studies. | Differences between studies. |
| Tracy and Wallace, 2016 (USA) | No | Systematic Review of effectiveness of peer support groups (one aspect of peer support services) in the treatment of addiction | Searched PubMed and MEDLINE. Included studies with adult participants, substance use, attending peer group support, RCTs or quasi experiments(pre-post data), US conducted from 1999 or later. | 10 studies included, N of participants within them ranging from 13 to 4420. | Revealed articles that support the use of peer support services within addiction treatment that address: 1)substance use, 2)treatment engagement, 3)HIV/HCV risk behaviours, 4) secondary substance related behaviours. | Peer provider (e.g. certified peer specialist, peer support specialist, mentor, recovery coach) - a person who uses his or her lived experience of recovery from mental illness and/or addiction plus skills learned in formal training, to deliver services in behavioural health settings to promote mind-body recovery and resiliency | No | No but studies included: ‘substance use addictions: alcohol, tobacco, legal/illicit drugs, prescription drugs’. | Varied across the 10 studies. | N/A |
| Eddie et al, 2019 (USA) | Yes - peer recovery support services (PRSS | Systematic Review of literature of effectiveness of PRSS in substance use disorder | Searched: PubMed, EMBASE, CINAHL, and PsycInfo | 6544 across 24 reports from 23 original studies (seven RCTs, four quasi-experiments, eight single- or multi-group prospective or retrospective studies, and two cross-sectional investigations) | PRSS may have particular utility in hospital/clinical outpatient settings. PRSS may be especially beneficial in substance detoxification units, and have the ability to improve outcomes for individuals engaged in inpatient or outpatient psychiatric treatment for SUD and co-occurring mental disorders. | ‘PRSS is a peer-helping peer service alliance in which a peer leader in stable recovery provides social support services to a peer who is seeking help in establishing or maintaining their recovery (SAMHSA, 2009). ‘Recovery coaches are peers trained to provide informational, emotional, social, and practical support services to people with alcohol or other drug problems through a wide variety of organizational sponsors, including recovery community centers, as well as hospital and outpatient clinical settings’ (White, 2009). | No | No | Varied between the 23 studies. | Typically paid employees working part- or full-time.Remuneration specifics not provided. |
| Davidson et al, 2010 (USA) | No | Review of literature on recovery support services (including peer-led) | Literature review but methods of searching not specified | Not specified | Recovery support services can play a variety of important roles in engaging people into care, supporting them throughout care/treatment and helping to achive better outcomes after. | ‘People who have overcome adversity can develop special sensitivities and skills in helping others experiencing the same adversity; this represents a “wounded healer” tradition that has deep historical roots in religious and moral reformation movements and is the foundation of modern mutual aid movements’ | No | Yes, DSM-IV | Various activities across studies mentioned, including peer outreach; peer and group based interventions |  |
| **PRIMARY STUDIES** | | | | | | | | | | |
| Ashford, Curtis and Brown, 2018  (USA) | Yes - Missouri Safe Project (MO Safe) | Quantitative, Cross-sectional, | Admin data analysis from clients' files; self-report; number of engagements | 417 drug injecting clients of the RCO model in Misouri. Age 35.59, male (58.5%) White (66.9%). 55.9% in stable housing, 34.1% had HCV, 38.1% currently on probation or parole. | 895 total peer engagements with 417 participants 35.7% had multiple engagements. Homeless people least likely to have MPE. | No (‘in recovery from substance use disorders) | 'Unstable housing' with categories: homeless, transitional housing, couch surfing | No | Both peer-based recovery support AND peer-led harm reduction services | Not specified |
| Bardwell, Boyd, Kerr & McNeil, 2018 (Canada) | No | Qualitative | Semi-structured interviews with service users and 30 hours of overt etnographic observation investigating the physical structures and layouts of 2 buildings (safe consumption sites), drug use practices within each shelter, and interactions between residents, peer workers and other staff. | 24 homeless injection drug users. Inclusion criteria required them to be residents on site and use drugs on site. | Residents described how the 2 shelters with harm reduction approaches were different to others, high barrier ones they used in the past and different from drug use in public spaces: sense of relief, less barriers and rules, normalizing drug use, being more at ease, feeling safe, judgment-free. Ongoing support from staff and peer witnesses. Collective sense of responsibility for preventing overdose. But having to negotiate space constraints across the shelters as not enough room or nor enough privacy, not sanitary.. | No | No | No | Peer workers were providing support, education, and overdose response | Not specified. Participants in interviews received $30 honorarium |
| Bardwell, et al., 2019 (Canada) | Yes, Tenant Overdose Response Organizers program (TORO) | Qualitative | Semi-structured interviews with tenants and 2 focus grouos with tenant organizers. 50 hours of etnographic observation in the buildings. | 20 tenants, 15 tenant organizers | Acceptability of TORO: 1) urgency and receptiveness - TORO timely and important; 2) enhanced knowledge, skills and recognition; sense of acknowledgment from other community members, sense of empowerment through having life-saving skills. Feasibility of TORO: 1) engaging isolated tenants; 2) environmental barriers to effective program implementation (physical conditions). Implementation opportunities and challenges: 1) community development - comfort in having a peer respond to overdose rather than emergency responders; less stigmatising and preferable. | Peer research assistants – ‘hired affected community members.’ | No | No | Peer research assistants conducted 50 hours of etnographic observation in the buildings; peer workers led the TORO program in their SRO buildings; peers in TORO underwent training in administering naloxone. | Peer research assistant hired but no specification on salaries. No other payment details provided. |
| Gonzalez et al., 2019 (USA) | No | Qualitative | Semstructured interviews with peer reentry specialists and with clients. | 7 peers + 3 clients | Peers reported caseloads between 3-16 clients. Who were typically discharged 8-9 months after they began working with their peer. Several peers perceived their role to be largely undervalued in their work environment. Program outcomes: most of peers’ time spent on helping clients obtain documentation. Recidivism connected to housing - some clients intentionally get arrested again to have a roof over their head. Housing identified as one of the most challenging services. | Peer reentry specialists - to qualify for employment as  a peer, an individual must have been at least 18 years old, had a mental health diagnosis or current / previous use of mental health services, be willing to use his or her own  experiences to help others recover, and completed a high school diploma or GED | No | No | Peer specialist provided prerelease in-reach, discharge planning, needs assessment,  navigation, and long-term relationship management among adults with a mental health  condition who needed community-based care upon release. | Peers were recruited, employed, and paid by a mental health service provider. Salaries not specified. |
| Barker et al., 2018a (UK) | Yes – Intentional Peer Support (IPS) | Qualitative | Semi-structured interviews about experiences of providing/receiving peer support and what were the critical factors to its success. | 29 | 6 main themes: 1) never give up; 2) experience-based relationships; 3) motivation; 4) overcoming obstacles; 5) how peers help (4 main ways: being role models, breaking boundaries, providing individualised treatment, and social support); 6) benefits for peers. | Definition of peer support - SAMHSA 'services [that] are delivered by individuals who have common life experiences with the people they are serving' who 'have a unique capacity to help each other' | Yes - 'single adults being without suitable accommodation including sleeping rough, in transient housing, or other inappropriate accommodation' | No | Often, participants are recipients of IPS before progressing into a peer supporter role; thus, experiences are reported from both perspectives of IPS providers and recipients. | No, volunteering. But received £10 voucher for participation. |
| Barker et al., 2018b (UK) | No | Mixed method. | Using Q methodology, which aims to objectively assess subjective viewpoints by statistically and qualitatively assessing a 'concourse' - communications sourrounding a topic of interest (Stephenson, 1953). | 40: 20 peers and 20 professionals | Analysis resulted in three differing viewpoints; the dominant viewpoint asserts that effective peer support is rooted in experiential knowledge, where peers build unique, trusting relationships to provide clients with a different level of support. | Definition of peer support - SAMHSA 'services [that] are delivered by individuals who have common life experiences with the people they are serving' who 'have a unique capacity to help each other' based on their shared experiences of phenomena such as mental health, addiction, and homelessness. Peer supporters were defined as peers by their organisations and usually had homelessness experience. Peers were currently working to provide support to homeless persons at various stages of recovery from homelessness. | No | No | Peer supporters were currently working to provide support to homeless persons at various stages of recovery from homelessness. | Not mentioned. |
| Bean, Shafer & Glennon, 2013 (USA) | Yes - Project H3 (Homes, Health, Hope) | Quantitative evaluation. | Structured surveys at move in day, 6 months after and 12 months after. Data on arrests provided from Pheonix Police Dep. | 47 but only 38 completed. | 98% participants remained in housing after 12 months. Statistically significant changes in particiapnts' report of substance use, quality of life and use of primary care physicians between baseline and 6 months. No statistical changes between 6-12 months. Arrest data showed statistically significant decrease in arrests from 12 months before to 12 monts after receiving housing. | 'Peer support uses specialists who have a history of homelessness, mental illness, or substance abuse and who are in recovery and offer supports to people who are not far along in their recovery' (Besio and Mahler, 1993) | No, but includes questions of where slept most frequently - streets, shelters, other | No, but includes questions: 'abused drugs/alcohol yes/no' and 'consumed alcohol every day for last month' yes/no and 'used injection drugs/shot' yes/no | The type of peer support the interviewed participants received from H3 was not specified. | Not mentioned. |
| Blondell et al., 2001 (USA) | Yes – comparing control group (usual care) with 2 types of intervention: 1) brief intervention (5-15 min physician delivered message) or 2) peer intervention (physican message + 30-60 min visit by ‘recovering alcoholic’) | Quantitative. | Retrospective nonrandomised intervention study with 3 arms. | 314 | 140 were contacted following hospital discharge; valid responses obtained from 45% of participants. Observed success rates for abstinance from alcohol since discharge were: 34% in control grouo, 44% in brief intervention and 59% in peer intervention; 36%, 51% and 64% at the 6th month following hospital discharge and 9%, 15% and 49% for initiation of treatment/self help. | ‘Recovering alcoholic’ - volunteers in the community who were active in AA. | No | Yes, DSN-IV plus positive toxicology for alcohol use disorder | Visiting hospitalised patients as part of their 12 Steps work. | No - volunteering |
| Boisvert et al., 2008 (USA) | Yes, Peer Support Community (PSC) program | Mixed-methods | Mixed methods including pretest/posttest measures, semi structured interviews and participant observation. | 18 in the PSC program, 7 relapsed, 1 graduated into other permanent housing thus 10 participants for the pre-post data. | Qualitative analysis: participants found: trust, respect, honesty, openness, helpfulness, leadership, integrity, willingness and sobriety important. Common goals identified were: shared experiences, giving hope, leading by example and teaching others. The purpose of the new community was: to stay clean, learn responsibility, be self-sufficient, create a safe environment, help others, provide a supportive environment and have an affordable community. Quantitative analysis: relapse rates for homelessness lowered from 85% 6 months prior to the study to 33% following institution of the PSC, and for SUD from 24% to 7%. | No | Classified as chronically homeless according to the US Housing and Urban Development guidelines | Chronic SUD but a minimum of 3 months sobriety prior to placement in the PSC programme. | PSC programme using occupational therapy guiding principles involving the use of occupation as a primary focus. PSH community attended meetings with the occupational therapist and discussed the principles of a peer-support recovery community or peer-driven community and a schedule of support meetings was arranged. Handbooks on the development of a peer support recovery community were provided to enable participants to form their own community’s mission statement and rules of conduct. | Not mentioned. |
| Charron et al., 2018 (Canada) | Yes, Participatory Research in Ottawa: Management and Point-of-care for Tobacco Dependence (PROMPT) | Mixed-methods. | Questionnaires before and after each of the 6 training sessions + interview with 2/4 of the most community- involved peers | 4 peers | From the interviews 3 themes: 1) personal gains and challenges with project leadership: (e.g. increase in self-confidence, but challenge was power issues between academic and peer researchers); 2) perception of community gains and challenges (e.g. peers did not expect as many of the study participants to reduce or quit tobacco, but issues of stigma in own community; 3) insights about project success and challenges –(e.g. peers felt accountable as project leaders). | Four community researchers were selected from the study target population comprised of current or previous tobacco and poly-substance users, who were homeless or at-risk for homelessness and resided in the inner-city region of Ottawa. Candidates were selected based on their keen interest in developing research in their community, previous experience as well as communication and networking skills. | No, but ‘homeless or at risk of homelessness’ | No | Peers involved in: study design, development of questionnaires, participant recruitment, administering consent form and questionnaires, as well as hand held spirometry after rigorous training. | Yes, paid an honorarium of $15 per hour for attending all training sessions, for a total of 48 h. |
| Collins et al., 2019 (USA) | Yes, HART-A (Harm reduction treatment for alcohol) | Quantitative | The 3-month efficacy of (HaRT-A) HaRT-A was developed using a three-phase community-based participatory research approach (CBPR). First phase – interviews with 50 people with lived experience of homelessness and AUD on their perspectives on available treatments + suggestions for improving treatment; second phase, a community advisory board to co-create the treatment based on the phase 1 findings; third phase - a 2-arm RCT to test HaRT-A’s initial efficacy. | 169 | Compared to control participants, HaRT-A participants reported signiﬁcantly greater increases in conﬁdence to engage in harm reduction and decreases in peak alcohol use, alcohol-related harm, AUD symptoms, and positive urinary ethyl glucuronide tests but findings were inconclusive regarding group diﬀerences on QoL (p > .12). | People with lived experience of homelessness and AUD | Defined by the McKinneyVento Act (The McKinney-Vento Homeless Assistance Act, 2009) for at least 6 of the last 12 months | DSM-5 criteria for psychiatric disorders (First, Williams, Karg, & Spitzer, 2015). The AUD portion of this measure documented the presence of AUD | People with lived experience of homelessness and AUD contributed to the development of the HART-A intervention. | Yes, $20 payment for participants; whether people with lived experience were paid for being part of the advisory board was not specified. |
| Crisanti et al., 2017 (USA) | Yes, HH Health Homes – a PSH (Permanent supportive housing) evidence based intervention for people experiencing homelessness | Quantitative | 2 groups - HH housed and not housed. | 237 at baseline, 152 completed the 6 month follow up interview, 98 the 12 month | Under the HH program 61.2% of participants received housing in the time period of the study. Improved overall health + decreased psychological distress in those who received housing vs those who just received services under the PSH model. Relationship between housing and overall health sig at 6 months follow up. | 'Peer support workers are people who self-identify as current or former clients of mental health and/or substance use services, have a period of demonstrated recovery (typically 2 years or more) and have completed specialized training in peer support services (Solomon, 2004). In this study also had experience of homelessness. | No, but . both unhoused and at risk of homelessness were included eg. Living at someone elses house, on the streets and in homeless shelters. | Yes, DSN-IV confirmed by face to face structured clinical interviews. | Peer-support workers (PSWs) were responsible for delivering case-management services. | Yes - employed but no other details provided. |
| Croft, Hayward & Story, 2013 (UK) | No, but all peers had experience of working at Find&Treat established to strengthen TB control in the socially excluded communities of London. | Qualitative | 60 minute semi structured interviews with a topic guide. | 6 Peers from Find&Treat, current or ex. Have had treatment for active TB and experience of homelessness and/or drug/alcohol dependecy within the last 3 years of the project. | Key themes: 1) making sense of the past, 2)renewed self - encourage empathy, inspire future opportunities, begins to identify with the peer role, learns tolerance; 3) the peer voice - unique power of the peer to reach the socially excluded; 4) project environment - talking about Find&Treat, community building. | Peer educators - Persons who have had TB and who have experienced homelessness and /or drug/alcohol dependency. | No | No | Peer education. | Yes, peer educators are in paid roles but no details provided. |
| Deering et al., 2011 (Canada) | Yes, the Mobile Outreach Project MAP | Quantitative. | Baseline and bi-annual follow up detailed semi-structured questionnaire administered by peer researchers plus HIV and violence questions asked by study nurse. | 249 female current or former sex workers. | 479 observations, 202 reported using the MAP between 2006-2008. Those who did access MAP were more likely to: have injected cocaine in the last 6 months, have more clients in the past week, solicit clients mostly in alleyways, have accessed inpatient addiction treatment/detoxification /residential drug treatment. Youth (less than 24 years) were significantly less likely to access the service. | Current/former sex workers who use drugs. | Unstable housing defined as 'not having a regular place to stay' | No | Peer researcher in the study and peer support worker in the MAP van but not much detail about their role. | Not specified. |
| Deering et al., 2009 (Canada) | Yes, a peer-driven intervention (PDI) developed to support uptake and adherence to highly active antiretroviral therapy (HAART) among women sex workers who use illicit substances in Vancouver. | Quantitative | PDI intervention had four key elements: weekly peer  support meetings, training for women to become  health advocates, a peer outreach  service, and drop-in onsite nursing service.Adherence to treatment was measured using pharmacy records, selfreport, and viral load outcomes. | 20 females currently or formerly involved in sex work, HIV positive. | The overall mean treatment adherence from pharmacy records was 87.9% per PDI-week (range, 26.2% to 100%). The amount by which viral load outcomes improved over the two time periods increased along with the number of PDI meetings attended, and women who attended 51–60 meetings had the highest increase (96%) in suppressed viral load tests over. | Health advocates -‘‘buddies’’, no definition provided. | No | No | Health advocacy – buddy system for women + outreach peer services | Participants were paid.. Outreach peer services had no mention of compensation |
| Ellison et al., 2016 (USA) | Yes, HUD/VASH (Department of Housing and Urban Development Veterans Health Administration Supportive Housing program + peer support | Quantitative | Randomised longitudinal, two site study. | First 50 veterans in the intervention arm of an intended sample of 90 participants. | Mean number of sessions that veterans engaged with peer supporters was 9 out of potential 40 but varied greatly between participants (0-39). The best predictor of engagement was time with most contacts occuring within the first 6 months. Older veterans had higher rates of engagement with peer supporters. | SAMSHA peer supporter 'a person who uses his or her lived experience of recovery from mental illness and/or addiction plus skills learned in formal training, to deliver services in behavioural health settings to promote mind-body recovery and resiliance' veteran with similar history of prior homelessness, substance use and mentasl illness but in recovery. | No | No | At each site the intervention consisted of meetings between veterans housed in HUD/VASH and full time trained veteran peer supporter | Not specified. |
| Goldade et al., 2012 (USA) | No | Quantitative | Cross sectional survey. | Participation rate was 90%. Total sample 4570 homeless people. Total who responded to the smoking questions was 4534. Out of the 4534 who completed smoking questions 12% (n=487) were former smokers, 70% current smokers, 14% never smokers and 4% refused to answer. | The significant predictors of willing to help were: sex (males more willing to help); age (older age); race/ethnicity with African Americans more willing to help; number of people known who have quit smoking (the higher the number of quitters known the more willing to help the person was) and also whether or not the person received social services as an adult. | Curently homeless ex-smoker. | Homelessness variables used: where did you sleep last night (emergency shelter, transitional housing, other)? Counting last night how long did you stay there? During your entire life how many different times have you been homeless? | No, but survey asked about age of smoking initiation and cigarettes per day when last smoked. | Taking part in a survey - to establish if currently homeless ex-smokers could be used as peer helpers to homeless smokers by asking who and how many of the ex-smokers would be willing to help. | Not specified. |
| Hunter & Power, 2002 (UK) | Yes, *Big Issue* peer education intervention | Mixedmethods | Participatory research design; questionnaire survey to investigate health promotion concerns of homeless people in London; focus groups and interviews with vendors and staff. | 10 vendors attended peer education training in Brighton, and 5 in West Midlands. Staff - 3 in focus group + 1 individual in London, 2 staff in individual interviews in each of Brighton and West Midlands | 1) preventing drug related harm including dealing with overdose was found to be important information need; 2) vendors had substantial opportunity for contact with the target group; 3) key themes from interviews: financial incentive was important for participation - vendor involvement in peer intervention would not be possible unless lost vending costs reimbursed, life experience as qualification for peer intervention; scepticism that other drug users will take the peers' advice despite 'peer status'. | Paper talks about the lack of definition in general - 'lack of theoretical base for peer intervention and the need for clarity about what exactly the process entails'. In this study potential peers are Big Issue vendors | Big Issue vendors including rough sleepers, those living in hostels, squats, traveller sites or B&B. | No | Vendors were involved in identifying health promotion needs of the homeless people; extent of vendors contact with other homelss people was examined; 3) 2 pilot peer education training sessions; 4) post training interviews with participants to explore the potential of the foundation to conduct a peer intervention. | Yes, £35 for attending a session (an amount suggested by Big Issue staff as recompense for lost  earnings). |
| Jozaghi & Reid, 2014 (Canada) | No | Qualitative | Semi-structured interviews with peers covering 6 themes: experience as peer worker, injection of their clients, overdose risk reductions, drug use culture, harm reduction eductation and an open discussion about anything raised during the interview. | 32 peer injection drug users | Activism - references to housing; fighting to get extra funding and support for safe injection and other harm reduction facilities; fighting to change people's perceptions of drug users. Needle distribution - peers believed there has been a reduction in risky drug use behaviour. Overdose - belief that supervised injection facilities and needle exchange facilities reduce the risk of overdose death. Education - role of peers in providing education resulting in reduced mortality and HIV and changed culture. Relationships - relationships that promote access to drug treatment services, sense of community. Change in behaviour - for some peers it helps to abstain from their own drug use. | 'Peer injection drug users' Employed (volunteering!) to provide coffee and juice; socialize with users and watch for signs of overdose. Some also work as peer counsellor. 8 hour shifts, small volunteer stipend. Those working at the 'chill lounge' must be current or former users themselves "to better understand, connect, and intercat with clientele who may be homeless, HIV positive and/or suffer from drug psychosis' | No | No, but the study is only about those injecting drugs | Provide coffee and juice; socialize with users and watch for signs of overdose. Some also work as peer ccounsellor. | Small volunteer stipend. |
| Kennedy et al., 2019 (Canada) | Yes, a peer staffed Overdose Prevention Site (OPS) facilities in Vancouver | Qualitative | Rapid ethnographic study. 185 hours of observational fieldwork between Dec 2016 and April 2017 + in depth qualitative interviews with 72 participants. | 72 | 1. OPS implementation and operations drew on existing community capacities - peers took up roles that were an extension to what they were already doing in the community; 2. peer workers foster environments of comfort and safety at OPS; 3. peer workers enable harm reduction practices; 4. there are benefits to the peer workers but also challenges | 'People who use(d) drugs, peer researchers - team members with research experience who currently use or have used drugs | 'Unstably housed = currently living in single occupancy hotel, shelter, homeless or having no fixed address' | No | Peer workers had training in naloxone distribution and their roles were an extension of what they already were doing in the communit. Peer researchers were involved in recruitment and interviewing. | Underpaid. |
| Lennox et al., 2017 (UK) | Yes, ENGAGER (pilot) vs treatment as usual | Mixed methods - pilot | Parallel 2 group RCT design, ENGAGER or treatment as usual (TAU) with parallel mixed methods process evaluation. | 60 male prisoners currently serving a prison sentence of up tp 2 years, within 4-16 weeks of release date | Recruitment aim of 60 people was reached in 9 months. Nearly half of all participants were in unstable accommodation in the 3 months prior to going to prison. Overall retention rate was 73% at 1 month and 47% at 3 month. Intervention was delivered to 36/40. 28/36 met with their practitioners in the community following their release. | Peer researchers - people with lived experience | No but ‘unstable accommodation included temporary accommodation, sofa surfing or homelessness’ | No | Involvement in the development of the intervention + peer mentoring but not much detail on this. | Not specified. |
| MacLellan et al., 2017 (UK) | Yes, part of HALT: Hepatitis study, a randomised controlled trial of a peer intervention for improving patient engagement with National Health Services across London. | Qualitative | Single narrative interview x 5 | 5 males with recent experience of homelessness accompanied by substance misuse and mental health challenges. Some had experience of Hepatitis C infection | 3 main techniques used to achieve 'connectedness' through establishing a positive therapeutic alliance with clients: 1) rapport, 2) self-disclosure, 3) shared group membership with health services. | Peer advocates (PA) - 'Individuals with previous patient or challenging social experiences'. had experienced homelessness in their recent histories accompanied by substance misuse and mental health challenges.. | No | No | PA role was to engage with referred clients, support and advocate for them through the appointment process and generally within peripheral services. PAs were invited to participate in the study at fortnightly peer meeting within the organisation at which they were based. | Not specified. |
| McCarthy et al., 2018 (USA) | Yes, peer specialist-led intervention for veterans with both a structured recovery model and unstructured support: Administering MISSION-Vet using Peer Support (AMPS) | Qualitative | Open-ended interview schedule for each of the 3 groups (veterans, peer specialists, case managers) with parallel questions; conduced after the intervention had concluded. | 20 veterans (16 high engagers, 4 low engagers); 3 peer specialists, 8 case managers. | Structured supprt: themes 1) flexibility; 2) the AMPS workbook used in all 20 scheduled structured sessions some veterans viewed it as homework. 3) time: 9-month duration with weekly visits of about 1 hour, not long enough - developing a solid relationship with a peer specialist takes time. Unstructured support: themes: reduced isolation, community reintegration and recovery activities. Satisfaction high with both peers and the AMPS program as a whole. | 'Peer specialist is an individual who is in recovery from a serious mental illness and has been hired as an employee to provide services for others with serious mental illness' (Davidson et al, 2006). Here all were also veterans themselves. | No | No | Peer specialists work with veterans in a Veterans Affairs homelessness program and were trained to deliver a recovery support program carried out weekly over 9 months including 20 structured sessions using a workbook and 20 unstructured, individually planned sessions. | Yes, it specified ‘employed’ but no details provided. |
| Mitchell et al., 2017 (Canada) | Yes, The BC Take Home Naloxone (THN) program implemented by the BC Centre of Disease Control (BCCDC) | Qualitative | 2 small focus groups and 5 individual interviews | 11 from the intensive case management; aged 19 - 25, self-identified as using opioids, received THN training by the Inner City Youth Program (ICY) in Vancouver. | Major themes that emerged were: perceptions of risk, motivations for participating in training; altruism, strengthening relationships and the importance of accessible naloxone (at the time of the study only available on prescription); empowerment and suggestions for youth-friendly improvements to THN education and service provision e.g. offering repeat and refresher training. | No, but here specified as 'with lived experience of THN, recruited from the young adults attending ICY.' | No, but specified as 'homeless or precariously housed' | Yes, defined by the American Psychiatric Association DSM 5 | Peer researchers co-conducted the focus groups and interviews and were trained in research methods and involved in all aspects of the study, including preparing interview schedules and data analysis. | Not specified. |
| Nyamathi et al., 2001 (USA) | Yes, a peer mentored vs nurse case-managed vs standard care HIV risk reduction program | Quantitative | Participants randomised by shelter. Measures of: cognitive resources; psychological resources; behavioural risk (drug and alcohol use, sexual activity, sociodemographic characteristics) at baseline and 6 months follow up. | 948 homeless women and their intimate partners at baseline (258 in peer, 360 in nurse case managed and 330 in standard care). 6 month follow up data from 633 (325 women = 69% of total n of women and 308 partners = 65% all partners) | Modest to marked improvements among the women and their partners can be seen for all 3 groups. Significant changes over time were found for all outcomes apart from self-esteem (significant improvements in nurse group only). Depression lessened in peer group and standard care but not in nurse group. Hostility decreased in peer group only. | 'Peer mentors' - 'individuals who are respected and recognised as natural helpers, educators and role models (Dearing et al, 1998). | 'Spent the previous night in a shelter, hotel, motel, or home of a relative or friend, and was uncertain as to her residence in the next 60 days or stated that she did not have a home or house of her own in which to reside (Gelberg &Linn, 1989). | No | Conducting 2 hr session weekly for 6 weeks, info on HIV/AIDS, risk behaviours, risk reducing and health protecting behaviours, condoms, condom placement skills, education session etc. | Not specified. |
| Poland et al., 2002 (Canada) | Yes, developing harm reduction materials by peers for others using drugs e.g. harm reduction video | Mixed methods | Participatory process. Process evaluation methods included in-depth individual interviews, focus groups, participant observation, and session debriefing forms. Summative evaluation research included focus testing of the harm reduction video and a survey of video users. | 60 participants + 6 peers | Youth developed a more than working relationships with each other, more like friends and family. Lessons learnt - perhaps too much control given to the youth, too many demands on them, limited time and resources. Video on harm reduction 'guide to fun and safe drug use' was seen as controversial by some senior management | No, 'street involved youths' hired on part time basis as peers. | Street-involved 'homeless youth as well as those who may be housed but who spend a significant portion of their time actively engaged in street culture'. | No | 6 street involved youths hired for 8 months to develop harm reduction materials for other street involved youth. they conducted focus groups and follow up one on one interviews + produced a 20 min video. | Yes. |
| Rayburn & Wright, 2010 (USA) | Yes, AA/12 Step | Qualitative | Semi-structured / largely unstructured interviews spanning 6 months. | No n reported. ‘Homeless alcoholic men in recovery program First Steps housed inside the Men's Pavilion’ - a shelter for homeless men supported by the Coalition for Homeless of Central Florida Inc | People adapt AA norms to suit their own life situations and these adaptaions ultimately facilitate the recovery. The adaptations are: exaggeration of the giving back to society; wanting to help other homeless alcoholics, volunteer at the coalition. exaggerated life aspirations/hopes for the future - ideal dream of perfection. | No, but men in recovery from alcohol use disorder in AA/Twelve Steps. | No | No | Twelve Step principles of giving back to the community and helping other ‘alcoholics’ | No |
| Stagg et al., 2019 (UK) | Yes, RCT of efficacy of peer support intervention for promoting engagement with health services for people with chronic Hepatitis C vs standard care | Quantitative | RCT non blinded controlled trial, 1:2 standard care to intervention arm. | Out of the 364 who consented 136 had a positive point-of-care test for HCV, 3 for HBV and 3 for HIV. 101 were enrolled, 63 randomised to treatment and 38 to standard care. | 50.5% of HCV positive individuals 51/101 engaged with services at least once in 6 months. 29.7 (30/101) achieved the sucessful outcome (3 engagemenst or more). Of the 30 7 wwere in control arm (7/38, 18.4%) and 23 (23/63) in the intervention arm (36.5%). In the model of absolute differences the intervention arm had an 18.1% (p=0.04) increased likelihood of a sucessful treatment outcome vs those in control group. | 'Peers have personal experience of a specific illness or lifestyle that enables them to support others experiencing similar challenges'. | No | No | In the community-controlled model of peer support,  participants in the intervention arm were individually  assigned to a peer advocate from the London-based  homeless charity and advocacy organisation Groundswell. | Not specified. |
| Tracy et al., 2012 (USA) | Yes, pilot of Mentorship for Alcohol Problems (MAP) | Mixed method | Stage 1 pilot: pilot/feasibility testing, manual writing, training program development and adherence/competence measure construction. Uncontrolled pilot, 10 mentors participated for 6 months until 30 mentees received MAP for 12 weeks. 4 focus groups with MAP participants | 40 (4 dropped out later). 30 mentees and 10 mentors. | Frequency of alcohol, drugs and both drugs and alcohol reduced significantly from baseline to week 12. All mentors remained abstinent from drugs and alcohol apart from 1. Strong positive response from participants re impact of MAP on their lives and its structure helped towards becoming or remaining abstinent, managing or reducing psychiatric symptoms reaching work related goals etc. | Mentors had to meet lifetime diagnosis for a substance use disorder (SCID-I) and were at least 6 months abstinent from drugs and alcohol. | No | Lifetime diagnosis for a substance use disorder (SCID-I) | Mentorship training, mentorship group (weekly, with mentors, mentees and clinician), individual mentorship contact (outside the group) | No |
| Tracy, Guzman & Burton, 2014 (USA) | Yes, Mentorship for Alcohol Problems (MAP) | Mixed methods | Association between participant characteristics and abstinance rates; and treatment processes and abstinance rates. Use of standardized measures, +. 4 focus groups conducted with participants. | 40 (4 dropped out later). 30 mentees and 10 mentors. | None of the participant characteristics were associated with abstinance. Supports the use of MAP for broad range of mentees. The 3 treatment processes associated with abstinance were: mentor supervision group attendence; mentee mentorship group attendence and metor mentorship group attendece, however the amount of time spent together as a pair on mentoring activities did not have any association on abstinance. | Mentors had to meet lifetime diagnosis for a substance use disorder and were at least 6 months abstinent from drugs and alcohol. | Yes - "not having housing/being declared homeless at any point during participation within the study" | Current diagnosis of a substance use disorder, actively using substances and recruited during the first 3 months of treatment when vulnerable to relapse | 2 cohorts of 20 participants. for each cohort 5 mentors engaged in mentoring activities for 24 weeks (6 months) until 15 recently admitted mentees participated in MAP for 12 weeks. | No |
| Tsai and Rosenheck, 2012 (USA) | Yes, Group Intensive Peer Support (GIPS) model of case management in a supported housing program for homeless veterans | Quantitative. | Pre-post nonequivalent cohort study that compared outcomes, service delivery and timing of housing acquisition. | Demonstration site 1 year before GIPS (n=102) and after (n=167). Other clients across other HUD-VASH sites across the country before (n=9,659) and after (n=21,318) implementation of GIPS at demonstration site. | Significant time x cohort interaction effect revealed that over the course of 6 months clients enrolled in GIPS showed a greater increase in social quality of life scores than clients at other sites but no other sig differences between sites or any sig site x cohort interactions related to housing, employment, income or clinical outcomes. so GIPS can be as effective as intensive community management. | No | No | No | GIPS gives support during weekly 1 hour long group meetings led by case managers where clients serves as active peers. | Not specified |
| Pakhale et al., 2018 (Canada) | Yes, the participatory research in Ottawa, management and point-of-care of tobacco (PROMPT) | Mixed-methods | Feasibility mixed methods community-based participatory action research. | 80 participants + 4 peers enrolled in the Smoking Treatment for Ontario Patients (STOP) programme led by the Centre for Addiction and Mental Health, Toronto Branch. | The 6-month follow-up rate was 42.5%. The baseline mean daily cigarette use was 20.5 and 9.3 cigarettes at study end, with mean reduction of 11.2 cigarettes at 6months (p<.001). There was a considerable reduction in self-reported illicit substance use plus psycho-socioeconomic benefits such as improved health, return to work and greater community engagement. | Community researchers - Individuals with lived experience, members of the PROMPT target population (ex/current tobacco smoker,  homeless or at-risk for homelessness, polysubstance user). | No, homeless or at risk of homelessness | Have used drugs in the past year excluding marijuana or alcohol | Weekly peer-led life skills workshops were conducted on a variety of topics such as financial literacy, banking, peer-support, HIV/hepatitis C education, pet-care, cooking, mindfulness meditation and art. Community researchers took the lead in organising and conducting these workshops. | Participants were given CAD$20 at baseline  enrolment and CAD$25 at each monthly visit to compensate for their time and effort but does not specify if community researchers were compensated also. |
| Weeks et al., 2006 (USA) | Yes, Risk Avoidance Partnership (RAP) peer-led intervention | Mixed-methods. | Ethnographic observations of the field training sessions and responses on the closing interview regarding the immediate impacts of the traiing and intervention program on the peers themselves. | 176 received intake interview, 130 (73.9%) initiated the training program. | Positive outcomes. Intervention was acceptable to the participants and trainig sufficient for the peers to deliver the harm reduction education/intervetion. | 'Peer/public health advocates' active drug users in peer-led intervention at sites that they and their peers use illicit drugs. | No | Active drug users (heroin / cocaine/crack) in the past 30 days | RAP peers trained to deliver standard harm reduction approaches e.g. condom distribution. | $25 for each interview (intake, closing, 6 month and any in-depth interviews) |
| Resnick and Rosenheck, 2008 (USA) | Yes, Vet-to-Vet peer education and suppoprt intervention vets with severe mental illnesses many of whom are also homeless | Quantitative | Quasi experiment. Control vs treatment exposed groups, 2 cohorts. Follow up interviews at 1 month, 3 month and 9 month after baseline assessment. Intention to treat analyses and as treated analyses. | 296: cohort 1 (n=78), cohort 2 (n=218). Those from cohort 2 who participated in more than 10 vet-to-vet groups in the period since last research interview formed cohort 2-v (n=102) | Compared with cohort 1 cohort 2 had sig higher 3 month follow up rates, and cohort 2-v had sig higher follow up rates at both 3 and 9 month. Within cohort 1 greater age and lower severity of trauma were both sig predictors of successful completion of follow ups. Primary outcomes: only general empowerment had sig group differences on both the as treated model and the intention to treat model. | Peer-provided sevices are not standardized in definition, terms include: consumer-operated services, peer support, mutual support and self help. Here 'people who have themselves experienced similar difficulties, learned to cope with them, and found reasons for hope for the future' | No | No | Peer - professional partnership model of peer education services. Meeting entirely peer-led Vet-to_Vet programs are affiliated and colocated with VA mental health services and VA mental health staff serve as consultants to the peer facilitators providing training. Ex veteran peer helped to design the study. | Not specified. |
| Groundwell report Finlayson et al., 2016 (UK) | Yes, HHPA Homeless Health Peer Advocacy program | Mixed-methods. | Anonymised Groundswell service use data; semi-structured interviews with nine current or former Peer Advocates | 9 current or former Peer Advocates. Groundswell data for 1,400 scheduled appointments across 285 individual clients in the year to 31st March 2015. | Multiple benefits: health of clients improved through: increased confidence, knowledge and motivation to access healthcare and to engage proactively with health management; decreasing reliance on unplanned secondary care services; and decreasing missed outpatient appointments. This results in 68% reduction in missed outpatient appointments, a 42% reduction in unplanned care activity, a saving of £2.43 for every £1 spent due to a reduction in unplanned care activity costs in the first 6 months following HHPA intervention. | Peer Advocates - experience of homelessness and some volunteering experience | Homelessness is classed as anyone who is registered as no fixed abode, anyone registered to a homeless hostel address in the London Boroughs of Lambeth, Southwark, Lewisham or Westminster, or anyone registered at The Dr Hickey Surgery or The Great Chapel Street Medical Centre. | No | Peer research approach for interviews with clients enabled rich and robust qualitative data to be collected. The peer researchers drew on their expertise to inluence the design of the research. | No, volunteering |
| Wright et al., 2006 (UK) | Yes, THN | Qualitative | Semi-structured interviews, topic guide based on housing and its impact on risk of overdose | 27 homeless drug injecting users - past or current history of heroin use and homelessness and either personal or peer experience of heroin overdose | Users were accurately able to identify signs of heroin overdose in peers. Respondents varied in their degree of prior knowledge re naloxone. Clear theme of willingness to administer THN in an emergency situation. | No | Yes, the lack of 'decent, secure, affordable home within a strong community' | No | Taking part in interviews regarding the feasibility of THN | N/A feasibility/exploratory research |
| Hayashi et al., 2010 (Canada) | Yes, peer-run outreach-based syringe exchange in Vancouver - Alley Patrol | Quantitative | Examined the rates of Alley Patrol Syringe Exchange Program (SEP) use throughout the study period - and looked at univariate associations between the explanatory variables and the use of SEP | 854 Vancouver based injection drug users from VIDUS (Vancouver Injection Drug Users Study) | In total 233 (27.3%) participants reported obtaining syringes from Alley Patrol volunteers at some point during the study period. Service use was associated with unstable housing, frequent heroin and cocaine use, injetcing in public and reusing needles. | No | No | No | Volunteers involved in SEP | Volunteering |
| Hirsch-Moverman et al., 2013 (USA) | Yes, peer based TB adherence intervention | Quantitative | RCT with intention to treat for patients offered latent TB intervention treatment at the Harlem Hospital Chest Clinic in NY, recruited into Tuberculosis Adherence Partnership Alliance Study | 252 (128 randomised to intervention and 124 to standard care) | 58.8% completed treatment. 60.9% in intervention vs 56.6% in control (ns). But treatment group was sig more likely to adhere to treatment. Currently homeless and currently using alcohol were sig predictors of not completing treatment over time. | Peers - 'Shared ethnicity, sex, illness experience, sexual orientation, risk behaviours and/or socio-economic characteristics with target population | No | No | Peer workers - completed LTBI or anti-TB treatment at Harlem Hospital and had  attended a 4-week training program that included role-playing exercises, informational  sessions and observation, which was designed to enhance their ability to provide social  support, information and instrumental support.’ | Not specified |
| Nyamathi et al., 2015 (USA) | Yes, intensive peer coaching + nurse case management vs intensive peer coaching only vs usual care | Quantitative | RCT for male parolees from prison or jail participating in a residential drug program (RDT), aged 18-60, with history of drug use prior to latest incarceration and homeless | 600 - 42 were screened out; 345 eligible for the Hep vaccine | No treatment differences were found on completion rates for the vaccine. History of injection drug use was related to vaccine noncompliance | Former parolees who sucessfully completed a similar RDT | Yes - one who does not have a fixed, regular, and adequate nighttime residence (National Health Care for the Homeless Council, 2014) | No | The peer coach interacted weekly for about 45 minutes with their assigned participants in person, and for those who left the facility, interacted by phone. Their focus was on building effective coping skills, personal assertiveness, self-management, therapeutic nonviolent communication (NVC), and self-esteem building. | Not specified. |
| Dechman, 2015 (Canada) | Needle exchange programs | Qualitative | 4 focus-groups with natural helpers/peers who help in needle exchange program SANE (the Sharp Advice Needle Exchange) in Cape Breton, Canada. | 12 in first 3 focus groups + extra 5 for the forth one | Peers may be the only connection many users have to any form of medical advice. They do whatever they can to help their friends – e.g. they let people who were homeless come to their homes for food, a shower, a night’s sleep. | Natural helpers, users who access needle exchanges who are recruited to act as secondary distributors | No | No | Secondary distributors of clean needles + extra help such as providing roof over head/food etc. First draft of the paper was also presented to the natural helpers for feedback. | 25 USD for time/travel per focus group; but in their roles as natural helpers referred to as volunteers with no mention of payment. |
| Ashford et al., 2019 (USA) | Yes, Rebel Recovery, a type of recovery community organisation (RCO) | Quantitative | Existing measures including demographics and unique identifiers | 396 consumers, using drugs, in Rebel Recovery | Consumers engaging at Rebel tend to experience high rates of homelessness, primarily use heroin have little to no fiscal resources and a small percentage reported being pregnant or involved in the child welfare system. It would appear that the Rebel program has been successful in engaging–at least initially–these underserved groups. | No | No | No | The study was designed with In line with CBPR with communities of PWUD and in recovery from SUD, peer leadership and staff from Rebel were involved in all stages of this study. Data collected by peer researchers. | Not specified. |
| Rosenblum et al., 2005 (USA) | Yes, service outreach and recovery (SOAR) | Quantitative | RCT with an intent to treat analysis. information and referral (I&R) + peer advocacy vs an experimental 12-session motivational group followed by a 36-session cognitive–behavioural group + I&R + peer advocacy | 289 indigent, residentially unstable adults  who frequented a large soup kitchen in NYC and reported  a history of drug and or alcohol problems | Soup kitchen attendees assigned to the low-threshold group counselling intervention, compared with those assigned to information and referral only, reported greater participation in substance abuse interventions and less alcohol use at follow-up. An unexpected finding was that the SOAR intervention significantly reduced alcohol use but not cocaine/crack use. | Peer advocates - were either in recovery from substance abuse or had been raised in drug- or HIV-affected families. | Residentially unstable | No | The peer advocates posted flyers, staffed a recruitment table inside the dining area of the soup kitchen, approached newcomers before and after meals, screened them, and briefly described the study to them. | Vouchers and modest  (US$ 5)  food coupon books or public transit passes |
| Bellamy et al., 2019 (USA) | Yes, Peerstar, LLC program | Mixed-methods | Primarily quantitative but supplemented by narratives from the forensic peer support staff as anecdotal evidence to demonstrate some of the benefits of forensic peer support staff | 77 peer specialists who provided peer support services to an active caseload of 429 individuals with SMI and/or co-occurring substance abuse disorders upon release from jail | In the first year after release, participants did much better than those in the general US prison population in terms of re-incarceration rates (21.7 percent vs 43.4 percent).While preliminary findings of this approach, this study reaffirms the idea that forensic peer support programs are beneficial in reducing recidivism rates for people diagnosed with a mental illness coming out of prison. | Forensic peer support: individuals that have their own experiences with navigating the criminal justice system as people with their own lived experiences of mental illness and/or addictions. | No | No | Providing peer support before and after release from prison | Not specified |
| Taylor et al., 2019 (Portugal) | Yes, mobile drug consumption room (MDCR) | Quantitative | Prospective client survey ahead of opening the first MDCR in Lisbon, Portugal | 72 prospective clients, reporting current injection drug use | 89% indicated that they were willing to use such a service. Among the 7% of participants who reported being unwilling to use the MDCR, the primary reason was already having a space to consume. The majority (75%) indicated a willingness to use the mobile unit every day. | No | yes ETHOS definition of homelessness | No but only involving injection drug users | Peer workers administered the survey to users and interfaced with community members during the fieldwork period. | Does not specify. No incentives for participation in survey. |
| Taylor et al., 2019 (UK) | Yes, data from The HALT: Hepatitis trial | Quantitative | A questionnaire captured self-reported  demographic and clinical information, including whether  participants had been vaccinated against HBV and how  many doses they had received. Participants were also asked to select a reason for not receiving  the vaccine or receiving less than three doses. | 346 hard-to-reach due to socio-structural factors that criminalise,  isolate and stigmatise who consented to participate in a RCT of a peer intervention to promote  engagement with hepatitis C services in London | 52.3% reported full HBV vaccination. Being female was associated with lower vaccine uptake. Intravenous drug use was associated with protection against incomplete HBV vaccination. The most common reasons declared for incomplete vaccination were never being offered the vaccine or not returning for further doses. | No | No | No | Participants are from a study captured earlier, that usues peers as outreach workers. | Not specified |
| Krawczyk et al., 2019 (USA) | Yes, Project Connections at Re-Entry (PCARE) | Quantitative | Intake interview at the mobile treatment van + treatment with buprenorphine/naloxone. Patients are referred by jail staff or can  walk in from the street. | 190 adults who are otherwise  disconnected from treatment and health services. primarily persons who are exiting jail or who have been recently incarcerated. | Of the 190 patients that received an initial buprenorphine prescription, 129 (67.9%) returned  for a second visit or more, and 60 (31.6%) were still engaged in buprenorphine treatment 30  days after initiation | No | No | No | A peer recovery specialist is available two mornings a week to work with patients to  address needs such as acquiring proper identification, enrolling in insurance, and connecting  with housing through linkage to partnering programs in the city of Baltimore such as ID  clinics and short and long-term shelter and housing programs. The peer meets with patients  during the intake process and later on a walk-in basis or by appointment to follow up on  specific patient needs. | Not specified |
| Hebert et al., 2008 (USA) | No | Quantitative | National survey of peer service delivery for veterans with mental illness, including those with comorbid substance use and homelessness | Not specified – small number | The number of participating programs was small. However, responses indicate that existing VHA peer services can be categorized as partnership services. Future areas of consideration include dissemination of peer support in VHA and other healthcare systems, with specific efforts to increase training protocols, evaluation, and payment options for peers. | Peer specialists have lived experience  of mental health issues, are in recovery, and are willing to  disclose their experience to assist others in earlier stages of  recovery. | No | No | Varies between organisations | Peers employed in the  Veterans Administrationhourly wages ranging from minimum wage to $20/h, and salaried positions from $16,600 to $34,920 per  year with benefits. |
| Latkin et al., 2003 (USA) | Yes, network oriented HIV prevention intervention | Quantitative | Randomised with an intent-to-treat model | 250 participants in the Self-Help in Eliminating Life-Threatening Diseases (SHIELD) study with at least weekly contact with drug users, willing to conduct AIDS outreach education, and be willing to bring into the clinic two network members for assessment | 92% returned at follow up. Experimental vs control 3 times more likely to report reduction in injection risk behaviours and 4 times more likely to report increased condom use. Participants in the experimental condition were more than 3 times as likely to report cessation of drug injection, almost 3 times as likely to report reduction in needle sharing, and over 7 times more likely to report increased condom use with casual partners | No | No | No | To help stop the spread of diseases such as HIV and Hepatitis in the community via peer outreach. | All participants were financially compensated for their time ($20 for baseline interviews, $25 for follow-up interviews, and $15 for group sessions). participants also received up to $25 for recruiting their risk network members for assessment |
| Stewart et al., 2009 (Canada) | Yes, a pilot  intervention study for homeless youth | Mixed-methods | One-group, within-subjects design examining the effects of the pilot intervention over time. Participants’ perceptions of impacts and satisfaction with the intervention were determined through qualitative interviews with the homeless youths. Quantitative data were elicited through standardised instruments for measuring intervention outcomes. | 56 homeless youth | The potential effects of this support intervention reveal encouraging trends, including expanded social network, improved emotional and mental well-being, decreased loneliness, acquisition of support-seeking coping and social skills, decreased use of drugs and alcohol, and adoption of healthier behaviours. | Peer mentors,including formerly homeless youths | Yes - considered homeless if they (1) had no home at all and were living on the streets (absolutely homeless); (2) were living in a place that was not intended as housing or was unsuitable for long-term residence; or (3) were at risk of becoming homeless through loss of their home, discharge from an institution/facility with nowhere to go, or loss of income. | No | 4 support groups, optional one-on-one support, group  recreational activities, and meals. Support was provided by professional and peer  mentors, including formerly homeless youths. | Following each interview, the youth was given a token of appreciation consisting of $20 in food vouchers, movie passes, and bus tickets. No mention of pay for the peer mentors. |
| **STUDY PROTOCOLS** | | | | | | | | | | |
| Parkes et al, 2019 (UK) | Yes, Supporting Harm Reduction through Peer Support (SHARPS) | Mixedmethod feasibility study with concurrent process evaluation | Peer-delivered, non-randomised relational intervention for people with problem substance use who are homeless or at risk of homelessness | 60 plus 4 Peer Navigators | N/A | ‘Peer Navigators’ are those with lived experience of homelessness and/or problem substance use who are employed as specialist support workers to provide emotional and practical support to individuals and help them to engage with relevant services. | No | Self-report alcohol or drug problems (with participants themselves recognising that their substance use is a problem for them | Peer Navigators will work with a small number of participants for up to 12 months providing both practical and emotional support | Yes - part-time (30 h per week, 18-month contracts) paid on a Specialist Support Worker rate. |
| Pakhale, Kaur, Florence et al., 2016 (Canada) | Yes, Participatory Research in Ottawa, Management and  Point-of-care of Tobacco (PROMPT) | Feasibility mixed methods communitybased participatory action research  project. | Prospective cohort study with community-based  participation and social network-based approaches to address tobacco dependence in hard to reach (homeless and with problemsubstance use) population in  inner city Ottawa | 80 plus 4 peers | N/A | Community members representing the study population' (ex or currently homeless, insecurely housed or multi drug users) | Homeless or insecurely housed | No | The peers have co-led all aspects of the project from  conceptualizing the study question to participating in knowledge translation | Yes -honorarium of $15 per hour (25 % above the  minimum wage in Ontario) for every hour spent working on the project |
| Swendeman et al., 2019 (USA) | Yes, automated text-messaging and monitoring (AMM) | Quantitative (RCT with 4 conditions) | Youth are randomized to: (1) enhanced standard of care of AMM and repeat HIV/STI testing assessment; (2) online group peer support via private social media plus AMM; (3) coaching available over 24 months, and delivered by near-peer paraprofessionals via text, phone, and in-person, plus AMM; and (4) online group peer support plus coaching and AMM. | 1500 (Youth aged between 12 and 24 years are being recruited from community-based organizations and clinics serving gay, bisexual, and transgender youth, homeless youth, and postincarcerated youth). | n/a | no | No | No | Patient navigation helping link people to health care and services, assist with insurance, problem solve barriers to care, and provide supportive counseling and follow-up to motivate engagement and retention in health and prevention services. | 'Personnel costs include hours and wages of staff to design and deliver the interventions, including peers, coaches, supervisors'. Does not specify. |
| Simmons et al., 2017 (USA) | Yes, feasibility of including peer support intervention together with HCRV (Health care for reentry veterans) | Mixed methods | Phase 1 (contextual analysis of reentry environment and resources in Massachusets) Veterans will each by interviewed 3 times, stakeholders once. Network map to show reentry services, processes for linkage and delivery of services and gaps in services. Phase 2 (implementation of the peer support)- health care utilization questionnaire, interviews with stakeholders and veterans, internal VA clinical and admin data. Phase 3 (Pennsylvania peer support implementation) similar to Massachusetts. | Phase 1: 10 veterans released from prison plus 20 stakeholders who currently assist reentry services, phase 2: 30 veterans for intervention +60 for comparison; phase 3: 10 veterans and 15 stakeholders for formative work and 30+60 veterans for the intervention. | Phase 2 and 3 outputs will include peer support guidebook and peer support training curriculum. | No | No | No | Doesn’t specify | Doesn’t specify |
| **COMMENTARIES** | | | | | | | | | | |
| Gardien and Laval, 2019 (France) | No | Commentary | Analysis of the institutionalisation process of the role of peer supporter in France, looking at 2 surveys. | n/a | Lack of clarity regarding peer role and disagreement re varius terms; inconsistent implementation. | Peer supporter - generic term encompassing: peer helper, peer counsellor, peer health mediator, peer worker, peer advisor.'previous experience of psychiatric treatment, drug addiction and living in precarious conditions (homelessness)' | Yes - living in precarious conditions | no | varies | Varies. minimal pay when employed within the HF context; voluntary (unpaid) status otherwise. |
| Power, 2002 (UK) | No | Commentary | Presentation of summary of three previously published studies on participatory research (1) process evaluation of a mobile needle and syringe exchange scheme, (2) peer-led intervention aimed at sexual health promotion targetting gay men, (3) health promotion needs of those experiencing homeless. | n/a | Participatory research can be done successfully and can be of value. | no | no | no | Varies between the 3 studies | Not specified |
| Bardwell et a., 2017 (Canada) | No | Commentary | Cmmentary on the need for more supervised consumption sites and other overdose prevention interventions across a range of housing sites to minimize overdose risk. | n/a | Safe consumption rooms work therefore surprising that they have not been rolled out more widely. Few interventions for PWUD who are unstably housed. | No | No | No | N/A | N/A |
| **CASE STUDIES** | | | | | | | | | | |
| Tookey et al., 2018 (Canada) | No | Qualitative | 2 case studies of transition/facilitators/challenges of moving from being a client to being a peer worker for drug using former Hepatitis C clients. Used interviews. | 2 (male 48 years old; female 50 years old) currently peers | 5 primary themes: 1) the role of prior experiences, 2) changes in substance use practices, 3) shifts in relationships with community members and friends, 4) supportive organizational and structural factors, 5) role transition as a journey. | Ex clients at the Toronto Community Hep C Program (TCHCP); who underwent Community Support Worker (CSW) training | No but both cases talk about living in shelters, on the streets, having unstable living conditions | No but both injection drugs users | Participatory case study design. Interviews were conducted with two current peer workers who were also involved in the study design, analysis and writing | Yes, peers are employees of South Riverdale Community Health Centre and paid an hourly wage, vacation pay and sick time.Not specified how much. |
| Chapman et al., 2018 (USA) | No | Qualitative | Comparative case study design to examine four US states with best practices in peer provider employment, via comprehensive site visits to study peer providers in mental health and SUD settings; expert panel amd interviews with: policymakers, training and certification specialists, peer providers, supervisors, and managers in peer-run, recovery-focused, and traditional treatment settings. | 194 individuals at 29 organizations were interviewed | An expert panel’s consensus was that Arizona, Georgia, Texas, and Pennsylvania were among the leading states in the employment and training of peer providers. Several key themes in the four study states have implications for the growth of peer provider employment nationwide: roles and job descriptions in various employment settings; training and certification approaches; billing and reimbursement for peer providers; workforce and career development; and maintaining recovery, addressing boundaries, and stigma | Peer providers are individuals who provide services in behavioral health settings—both mental health and substance use disorders (SUDs) treatment—based on their own experience of recovery from mental illness or addiction and skills obtained from formal peer provider training (SAMHSA-HRSA Center for Integrated Health Solutions) | No (and not a focus of this paper) | No | Varies | This paper compared the situation of peer workforce across 4 US states and found that if peers are being paid they are low waged and paid less than other staff and have less opportunities for growth and promotion. |
